# A gamma‐thionin protein from apple, MdD1, is required for defence against *S*‐RNase‐induced inhibition of pollen tube prior to self/non‐self recognition

**DOI:** 10.1111/pbi.13131

**Published:** 2019-05-17

**Authors:** Zhaoyu Gu, Wei Li, James Doughty, Dong Meng, Qing Yang, Hui Yuan, Yang Li, Qiuju Chen, Jie Yu, Chun sheng Liu, Tianzhong Li

**Affiliations:** ^1^ Laboratory of Fruit Cell and Molecular Breeding China Agricultural University Beijing China; ^2^ Department of Biology and Biochemistry University of Bath Bath UK

**Keywords:** Malus domestica, MdD1, pollen tube growth, plant defence, *S*‐RNase

## Abstract

Apple exhibits *S*‐RNase‐mediated self‐incompatibility. Although the cytotoxic effect of *S*‐RNase inside the self‐pollen tube has been studied extensively, the underlying defence mechanism in pollen tube in Rosaceae remains unclear. On exposure to stylar *S*‐RNase, plant defence responses are activated in the pollen tube; however, how these are regulated is currently poorly understood. Here, we show that entry of both self and non‐self *S*‐RNase into pollen tubes of apple (*Malus domestica*) stimulates jasmonic acid (JA) production, in turn inducing the accumulation of *MdMYC2* transcripts, a transcription factor in the JA signalling pathway widely considered to be involved in plant defence processes. MdMYC2 acts as a positive regulator in the pollen tube activating expression of *MdD1*, a gene encoding a defence protein. Importantly, MdD1 was shown to bind to the RNase activity sites of *S*‐RNase leading to inhibition of enzymatic activity. This work provides intriguing insights into an ancient defence mechanism present in apple pollen tubes where MdD1 likely acts as a primary line of defence to inhibit *S*‐RNase cytotoxicity prior to self/non‐self recognition.

## Introduction

Plant reproduction requires reciprocal recognition and communication events between the pollen and the stigma/style cells (Boavida *et al*., 2005; Higashiyama, 2010; Suzuki, 2009; Thomas and Noni, 2013). While many flowering plants exhibit intraspecific reproductive barrier that is widely found to prevent self‐fertilization in order to promote cross‐breeding, this process is called self‐incompatibility (SI) (Chen *et al*., 2018; Franklin‐Tong, 2008; Li *et al*., 2018; Nettancourt, 2001; Takayama and Isogai, 2005). To date, a number of genes involved in SI system between pollen and pistil have been identified, among these many plant species from the Rosaceae, Plantaginaceae and Solanaceae family exhibit *S*‐RNase‐based gametophytic self‐incompatibility (GSI), which is the most commonly occurring and has been centrally studied (Anderson *et al*., 1989; Broothaerts *et al*., 2004; De *et al*., 2012; Roalson and Mccubbin, 2003; Sassa *et al*., 1992, 1994). Apple (*Malus x domestica*), which belongs to *S*‐RNase‐based GSI in Rosaceae, is one of the most important horticultural crops in the world. In the GSI system, *S*‐RNase is female determinant. During fertilization, *S*‐RNase is transported into pollen tubes by ABCF protein and recognized by *S*‐locus F‐box protein (SLF/SFBB), which is the male determinant (Anderson *et al*., 1989; Hua *et al*., 2008; Mcclure, 2004; Meng *et al*., 2014; Qiao *et al*., 2004; Sijacic *et al*., 2004; Wei *et al*., 2014).

*S*‐RNases are style‐specific secreted ribonuclease glycoproteins, which act both as recognition proteins and as highly specific cytotoxins. The critical role of *S*‐RNase is the rejection of self‐pollen. In GSI response, both self and non‐self pollen tube could grow into pistil. In compatible pollen tubes, *S*‐RNase subsequently degrades via the ubiquitin‐26S‐proteasome pathway, while in incompatible pollen tubes, *S*‐RNase exhibits cytotoxicity characteristic and has been attributed to its degradation of pollen rRNA, resulting in the arrest of protein synthesis (Gray *et al*., 1991).

Recent studies in Rosaceae mainly focus on the downstream signalling cascade likely occurs in SI in order to terminate incompatible pollen tubes growth, such as changes of calcium concentration, actin cytoskeleton depolymerization, burst out of reactive oxygen and tRNA aminoacylation in pollen tubes (Di *et al*., 2010; Li *et al*., 2018; Wang and Zhang, 2011; Wang *et al*., 2009). Indeed, in general, when an extracellular protein enters a cell, a series of processes will be triggered, including defence response and signal transduction (Kwak *et al*., 2014; Sawamoto *et al*., 1996; Wan *et al*., 2009). In response to undesirable invading substances, plants could activate a wide range of defences which can reduce potential cellular damage (Erb *et al*., 2012; Schuman and Baldwin, 2015; Wu *et al*., 2010). Taken together, as *S*‐RNase possesses cytotoxicity, it seems that *S*‐RNase may trigger signalling pathways or defence responses following its entry into the pollen tube. Furthermore, SI and the plant innate immunity systems are considered to have common pathways, and Nasrallah (2005) proposed that SI originates from an ancient plant defence system so that there are many common pathways between SI and plant defence; for instance, programmed cell death is caused by RNase‐based cytotoxicity (Dangl and Jones, 2001; Geitmann *et al*., 2004; Jones and Dangl, 2006; Jordan *et al*., 2000; Nasrallah, 2005; Penn and Potts, 1999; Schuman and Baldwin, 2015).

Previous reports have demonstrated that cysteine‐rich peptides (CRPs), comprising various subgroups of defence molecules, regulate a wide range of reproductive processes and defence in plants, including pollen tube growth and guidance, gamete activation and pathogen responses (Bircheneder and Dresselhaus, 2016; Qu *et al*., 2015; Satohiro *et al*., 2009; Takeuchi and Higashiyama, 2016; Tavormina *et al*., 2015). Moreover, in GSI system of poppy (*Papaver rhoeas*), stigma S‐determinant (PrsS) encodes a secreted CRP, is a stigma determinant in poppies and could activate downstream signalling events when it interacted with incompatible pollen (Foote *et al*., 1994; Wheeler *et al*., 2010). In addition, *Brassica* families display another SI system, SCR, a S‐locus CRP cysteine‐rich peptide, that is a pollen determinant in *Brassica* and structurally similar to defensin (Kachroo and Nasrallah, 2002; Schopfer *et al*., 1999; Suzuki *et al*., 1999; Takayama *et al*., 2001). These findings propose that CRPs act as signalling substance triggering varies responses in SI process. Furthermore, previous studies have shown that defence genes, such as defensins or γ‐thionins, could be regulated by jasmonic acid (JA), which is known to be important for plant defence and fertility (Ahmad *et al*., 2016; Davis *et al*., 1969; Devoto and Turner, 2003; Farmer and Ryan, 1990; Ishiguro *et al*., 2001; Melo *et al*., 2002; MuradoğLu *et al*., 2010; Nakata and Ohme‐Takagi, 2013; Song *et al*., 2005; Thomma *et al*., 1998; Wasternack and Hause, 2013; Wasternack *et al*., 2013; Yildiz and Yilmaz, 2002; Yuan and Zhang, 2015). Thus, the SI reaction is more likely to trigger the defence response in pollen tube in Rosaceae species. To date, it has not been reported that plant hormones or CRPs participate in pollen tube growth during the SI response in apple.

In GSI system in the Rosaceae, both self and non‐self *S*‐RNase could be transported into pollen tubes, and then, recognition occurs in the tube (Meng *et al*., 2014). However, there has been no evidence of the molecular events that occur between entry of *S*‐RNase into pollen tubes and its recognition. A key question is whether non‐self *S*‐RNase also affects pollen tube growth in a negative way before its targeted degradation? Are there endogenous response mechanisms which combat the effects of *S*‐RNase during the early stages of pollen tube growth in the style? Thus, we propose the feasibility that a plant defence response may be triggered by self/non‐self *S*‐RNase in the pollen tube. Here, we focus on the *S*‐RNase‐induced reaction in pollen tube before self/non‐self discrimination. We report that apple *S*‐RNase triggers the accumulation of JA in both self and non‐self pollen tubes, resulting in the deployment of defence protein, MdD1. We also provide evidence that the MdD1 protein inhibits *S*‐RNase activity by interacting with its active site, ensuring normal pollen tube growth prior to self/non‐self recognition via the SI mechanism. Based on these results, we propose that MdD1 acts as a primary defence protein in apple pollen tubes facilitating compatible pollen tube growth in the presence of *S*‐RNase.

## Results

### *S*‐RNase increases the concentration of JA in both compatible and incompatible apple pollen tubes

As the SI reaction primarily occurs in the pollen tube, we established an *in vitro* pollen tube culture assay to study SI in apple. For self‐pollination‐induced (SPI) treatment, equal amounts of recombinant *S*‐RNase proteins (*S*_*1*_‐RNase and *S*_*2*_‐RNase) matching the ‘*Ralls Janet*’ pollen *S* haplotype (*S*_*1*_ *S*_*2*_) were added to the pollen germination media at a range of concentrations. For cross‐pollination‐induced (CPI) treatment, equal amounts of recombinant *S*‐RNase proteins (*S*_*3*_‐RNase and *S*_*9*_‐RNase) were added to the pollen germination media at a range of concentrations. The results showed that pollen tube lengths were found to be significantly reduced when the total concentration of self *S*‐RNase (*S*_*1*_‐RNase and *S*_*2*_‐RNase) was 30 μg/mL. In contrast, pollen tubes were not significantly suppressed when the concentration of non‐self *S*‐RNase (combination of *S*_*3*_‐RNase and *S*_*9*_‐RNase) was 30 μg/mL. Furthermore, pollen tube growth was inhibited by both self and non‐self *S*‐RNases when these proteins were added at a concentration of 45 μg/mL, indicating that high protein concentration of *S*‐RNase could not distinguish self and non‐self *S*‐RNase treatment, while inactivated *S*‐RNase, which was denatured by boiling, failed to induce significant inhibition of pollen tube growth (Figure S1). This result clearly demonstrates that SI was induced by adding recombinant *S*_*1*_+*S*_*2*_‐RNase proteins to a final total concentration of 30 μg/mL in the pollen culture medium and that self and non‐self reactions can be distinguished *in vitro*.

**Figure 1:**
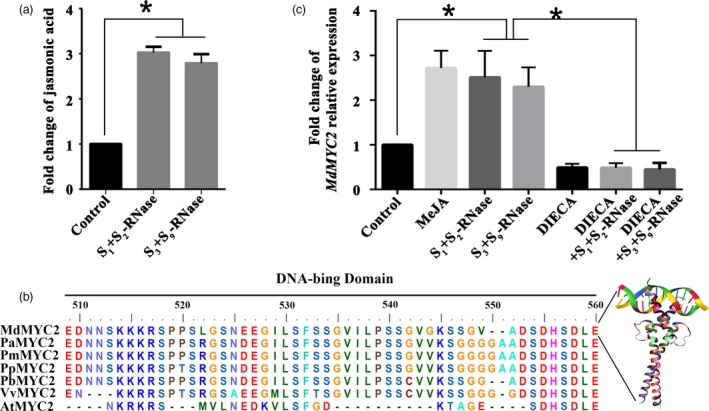
*S*‐RNase induces the JA‐MdMYC2 signalling pathway in pollen tubes. (a) Pollen tubes from ‘*Ralls Janet*’ were treated with 30 μg/mL of *S*_*1*_+*S*_*2*_‐RNase and *S*_*3*_+*S*_*9*_‐RNase, respectively, and then collected the pollen tubes. Fold change of jasmonic acid content in ‘*Ralls Janet*’ pollen tubes treated with 30 μg/mL of *S*_*1*_+*S*_*2*_‐RNase and *S*_*3*_+*S*_*9*_‐RNase. Control, untreated pollen tubes. (b) Comparison of the deduced amino acid sequence of MdMYC2 with MYC2 protein sequences from other plant species. The DNA‐binding domain is shown as a black bar above the alignment. AtMYC2 is from *Arabidopsis thaliana*, PaMYC2 is from *Prunus avium*, PmMYC2 is from *Prunus mume*, PpMYC2 is from *Prunus persica*, PbMYC2 is from *Pyrus bretschneideri*, and VvMYC2 is from *Vitis vinfera*. (c) Fold change of *MdMYC2* relative expression. qRT‐PCR analysis of *MdMYC2* expression in pollen tubes following various treatments. Pollen tubes were treated with 30 μg/mL of *S*_*1*_+*S*_*2*_‐RNase and *S*_*3*_+*S*_*9*_‐RNase, 30 μm methyl jasmonate 30 μm (MeJA), sodium diethyldithiocarbamate (DIECA) and 30 μg/mL of *S*_*1*_+*S*_*2*_‐RNase and *S*_*3*_+*S*_*9*_‐RNase after DIECA treatment. Control, untreated pollen tubes. The final data were normalized to the expression in the untreated pollen tubes (control). Values are means + SD of three biological replicates. Asterisks indicate significantly different values (*P <* *0.05).

To investigate whether self or non‐self *S*‐RNase influences JA content changes in pollen tubes, we used high‐performance liquid chromatography (HPLC) and observed a significant increase of JA levels in apple pollen tubes under the treatments of *S*_*1*_+*S*_*2*_‐RNase (self) and *S*_*3*_+*S*_*9*_‐RNase (non‐self) (Figure 1a). These results suggested that the JA content could be induced by both self and non‐self *S*‐RNase. And there was no significant difference in JA level between the self and non‐self *S*‐RNase treatments. Given that MYC2 has previously been identified as a key TF involved in the JA signal transduction pathway (Kazan and John, 2013), in order to further investigate the regulation mechanisms of JA response to *S*‐RNase in apple pollen tubes, we identified the *MdMYC2* cDNA sequence from RNA extracted from ‘*Ralls Janet*’ pollen tubes. *MdMYC2* is predicted to encode a protein with bHLH‐MYC TF N‐terminal structural domain (Figure S2). Moreover, based on protein sequence alignment and crystal structure superimposition analysis, MdMYC2 polypeptide is composed of helix–loop–helix domains, as is characteristic of the specific DNA‐binding proteins (Figure 1b, Figure S2 and Table S1). Combining the result of subcellular localization, MdMYC2‐GFP fluorescence indicated that MdMYC2 is localized in the nucleus (Figure S3b), indicating that MdMYC2 act as a TF in apple pollen tube. The expression of *MdMYC2* was evaluated in different organs of apple, the results showed that *MdMYC2* transcript abundance ubiquitously expressed in all organs (Figure S3a). In addition, qRT‐PCR revealed that expression of *MdMYC2* in cultured ‘*Ralls Janet*’ pollen tubes increases significantly after both self and non‐self *S*‐RNase treatments (Figure 1c). Furthermore, the expression of *MdMYC2* was also induced by MeJA treatment in pollen tube (Figure 1c), indicating that it participates in JA pathway. No difference was observed in the expression of *MdMYC2* between samples of pollen tubes that had been treated with the JA biosynthesis inhibitor, DIECA (sodium diethyldithiocarbamate), and samples of pollen tubes that had been treated with *S*_*1*_+*S*_*2*_‐RNase/*S*_*3*_+*S*_*9*_‐RNase after DIECA treatment (Figure 1c). These results suggested that the expression of *MdMYC2* may be induced in pollen tubes by both self and non‐self *S*‐RNase via the JA signalling pathway.

### MdD1 is a downstream factor in the MdMYC2 response to *S*‐RNase in apple pollen tubes

**Figure 2:**
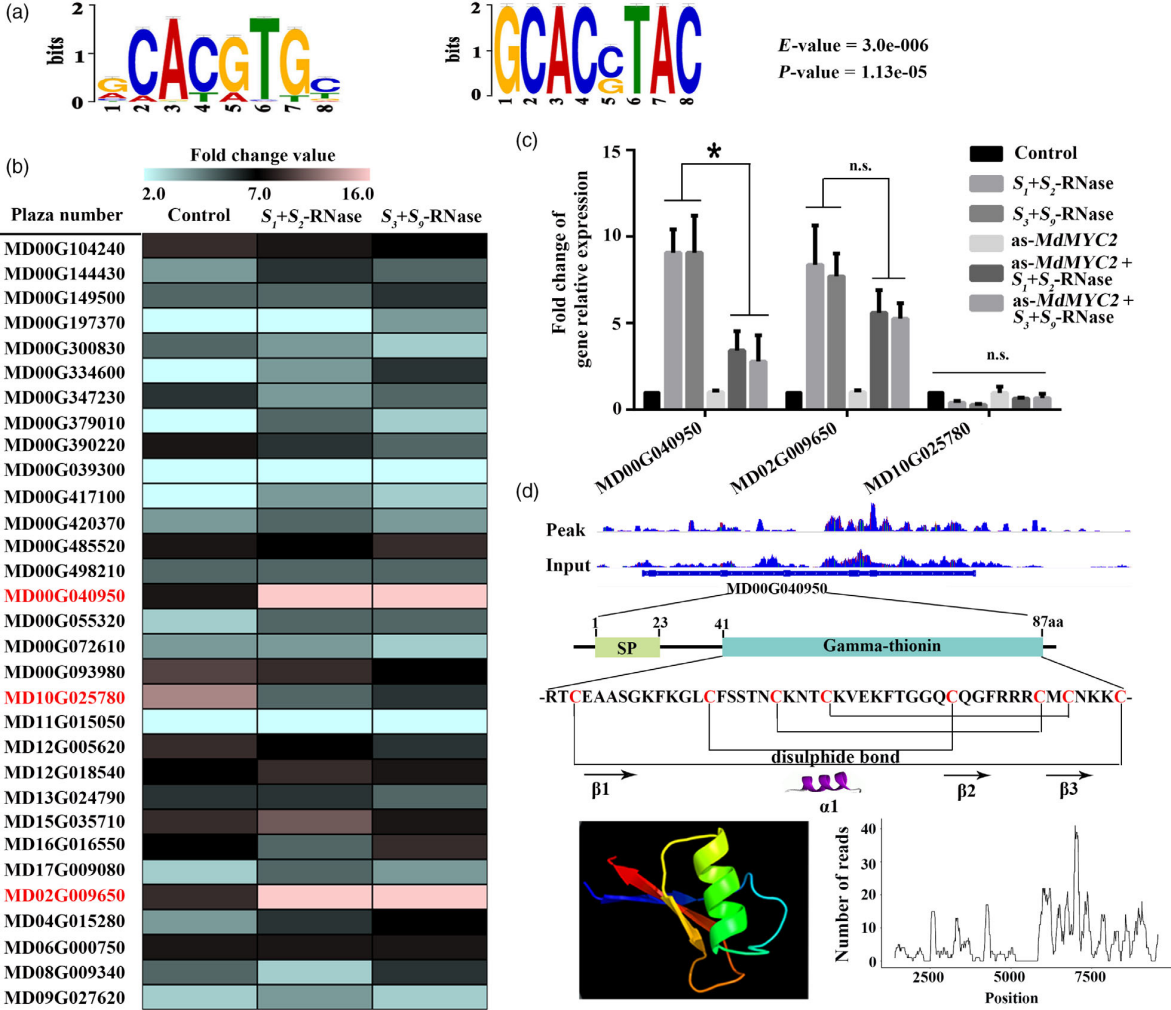
MdD1 is a downstream factor in the MdMYC2 response to *S*‐RNase in apple pollen tubes. (a) MdMYC2 binding elements. Potential MdMYC2 binding motifs as determined by MEME analysis. (b) The candidate gene expression levels were investigated by qRT‐PCR. Expression of candidate genes which expressed in pollen tube under *S*_*1*_+*S*_*2*_‐RNase and *S*_*3*_+*S*_*9*_‐RNase‐treated pollen tubes. Screening target genes, which strongly up‐regulated expression in pollen tubes, in response to both *S*_*1*_+*S*_*2*_‐RNase and *S*_*3*_+*S*_*9*_‐RNase. (c) Expression of MdMYC2 target gene levels was investigated by qRT‐PCR in *S*_*1*_+*S*_*2*_‐RNase and *S*_*3*_+*S*_*9*_‐RNase treated with or without *MdMYC2* silencing using antisense oligonucleotide *MdMYC2* (as‐*MdMYC2*). (d) Structure analysis of MdD1.

In order to identify the downstream gene targets of MdMYC2, we performed a chromatin immunoprecipitation sequencing (ChIP‐seq) assay using anti‐MdMYC2 antibody to pull down the genomic fragments bound by MdMYC2 *in vivo* in apple (Figure S4). Based on the binding pattern analysis, we found that G‐box is a MdMYC2 binding motif (Figure 2a). Using a distance to transcription start site between −1000 and +100 bp, a total of 114 candidate genes were identified, 30 of which were found to be expressed in pollen by qRT‐PCR (Figure 2b, Table S3). Using quantitative PCR, we identified three genes from the 30 pollen‐expressed candidate genes that responded to self or non‐self *S*‐RNase in a *MdMYC2‐*dependent manner (Figure 2b). Of these, a gene, in which plaza number is MD00G040950, exhibits strongly up‐regulated expression in pollen tubes in response to both self and non‐self *S*‐RNases. Furthermore, we silenced *MdMYC2* in pollen tube to analysis whether *MD00G040950* was regulated by MdMYC2. The result was shown that the expression was significantly down‐regulated when *MdMYC2* was silenced by antisense oligonucleotide (as‐ODN) (Figure 2c). Therefore, we chose *MD00G040950* for further research. Structure analysis showed that *MD00G040950* is predicted to encode a protein that has sequence homology to γ‐thionins, and to possess a signal peptide (Figure 2d and Table S1). Phylogenetic analysis of homologs from other species revealed the closest relationship between MD00G040950 and pear (*Pyrus*) defence proteins (Figure S5b), so we renamed the corresponding gene, MdD1. qRT‐PCR analysis showed that *MdD1* was ubiquitously expressed in most organs of apple, with the highest expression in pollen (Figure S5a). Particle bombardment of growing apple pollen tubes was performed with a construct containing the MdD1 fused to green fluorescent protein (GFP), and heterologously expressed MdD1‐GFP in maize protoplasts to investigate the subcellular localization of MdD1. In both cases, MdD1 was observed to localize mainly in the cytoplasm (Figure S5c, d).

**Figure 3:**
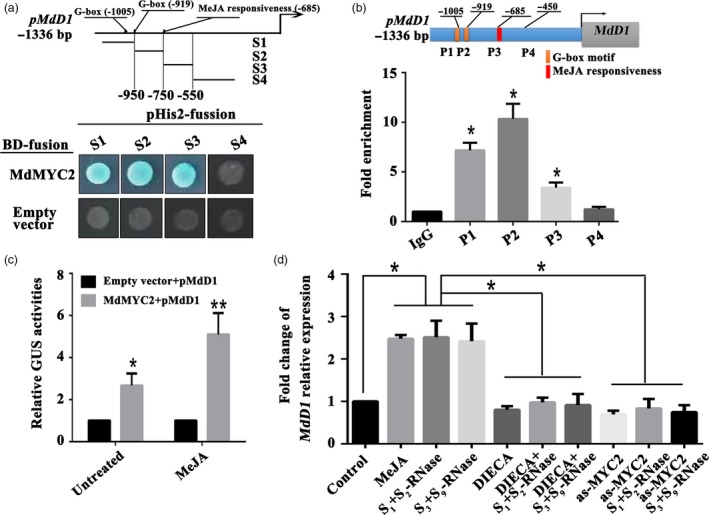
*S*‐RNase induces *MdD1* expression via the JA‐MdMYC2 signalling pathway in pollen tubes. (a) Yeast one‐hybrid (Y1H) analysis showing that MdMYC2 binds to the *MdD1* promoter fragment containing the G‐box motif and MeJA responsiveness motif. The promoter of *MdD1* was divided into four fragments (S1‐S4). S1 and S2 fragment contain G‐box motif, respectively; S3 fragment contains MeJA responsiveness motif; S4 fragment without the binding motif as a negative control. X‐α‐gal was used as a screening marker. The empty vector and the *MdD1* promoter were used as negative controls. These experiments were repeated three times. (b) Chromatin immunoprecipitation (ChIP)‐PCR showing the *in vivo* binding of MdMYC2 to the *MdD1* promoter. Chromatin samples were extracted from pollen tubes and precipitated with an anti‐MdMYC2 antibody. Eluted DNA was used to amplify the sequences neighbouring the G‐box by qPCR. Four regions (P1–P4) were examined. The ChIP assay was repeated three times, and the enriched DNA fragments in each ChIP were used as one biological replicate for qPCR. Data are the mean ± SEM. *P <* *0.05. (c) *β*‐glucuronidase (GUS) reporter activity analysis, showing that MdMYC2 activates the *MdD1* promoter and transcriptional activity was significantly enhanced under MeJA treatment. The MdMYC2 effector vector, together with the reporter vector containing the *MdD1* promoter, was co‐injected into tobacco leaves to analyse the GUS activity. The empty vector as control, together with the reporter vector containing the MdD1 promoter, was co‐injected into tobacco. Three independent transfection experiments were performed. Data are the mean values ± SEM. *P <* *0.05, **P <* *0.01. (d) Fold change of *MdD1* relative expression by qRT‐PCR. The following pollen tube treatments were used: methyl jasmonate (MeJA) and the inhibitor (DIECA for MeJA); a treatment with the above inhibitors followed by S‐RNase treatment; a treatment with *S*‐RNase with or without *MdMYC2* silencing. Pollen tubes without any treatment were used as controls. Data from three biological replicates were combined using a linear mixed‐effects model. The final data were normalized to the expression in the untreated pollen tubes (control). Data are the mean ± SEM. *P <* *0.05.

To confirm the regulation of the *MdD1* promoter by MdMYC2, MdMYC2 binding to the *MdD1* promoter was examined by yeast one‐hybrid (Y1H) analysis. Various fragments of the promoter were tested, and this revealed that MdMYC2 bound to a fragment containing the G‐box motif and a MeJA responsiveness motif (Figure 3a). To confirm that MdMYC2 bound to the promoter of *MdD1 in vivo*, a chromatin immunoprecipitation (ChIP)‐PCR assay was performed. The results showed that MdMYC2 significantly enhanced the PCR‐based detection of the *MdD1* promoter, indicating that MdMYC2 binds to the *MdD1* promoter *in vivo* (Figure 3b). In addition, using a GUS (*β*‐glucuronidase) transactivation assay in tobacco leaves involving co‐transformation with the 35S:MdMYC2 and pMdMdD1:GUS constructs, we saw that the activity of the *MdD1* promoter was enhanced by MdMYC2 and further enhanced by jasmonate (MeJA) treatment (Figure 3c). It was also observed that *MdD1* expression was up‐regulated by both self and non‐self *S*‐RNase treatments. The expression of *MdD1* was also induced by MeJA treatment in pollen tube (Figure 3d), indicating that it participates in JA pathway. No difference was observed in the expression of *MdD1* between samples of pollen tubes treated with DIECA and samples of pollen tubes treated with *S*_*1*_+*S*_*2*_‐RNase/*S*_*3*_+*S*_*9*_‐RNase after DIECA treatment, or between samples of pollen tubes that were *MdMYC2* silenced by as‐ODN and *S*_*1*_+*S*_*2*_‐RNase/*S*_*3*_+*S*_*9*_‐RNase treated after silencing *MdMYC2* (Figure 3d). Additionally, a sense oligonucleotide (s‐ODN) assay and *S*_*1*_+*S*_*2*_‐RNase/*S*_*3*_+*S*_*9*_‐RNase‐treated tubes after s‐ODN‐*MdMYC2* were run as controls (Figure S6). These results suggest that the expression of *MdD1* in apple pollen tubes is regulated by both self and non‐self *S*‐RNase through JA‐MdMYC2 signalling.

### MdD1 inhibits *S*‐RNase activity

**Figure 4:**
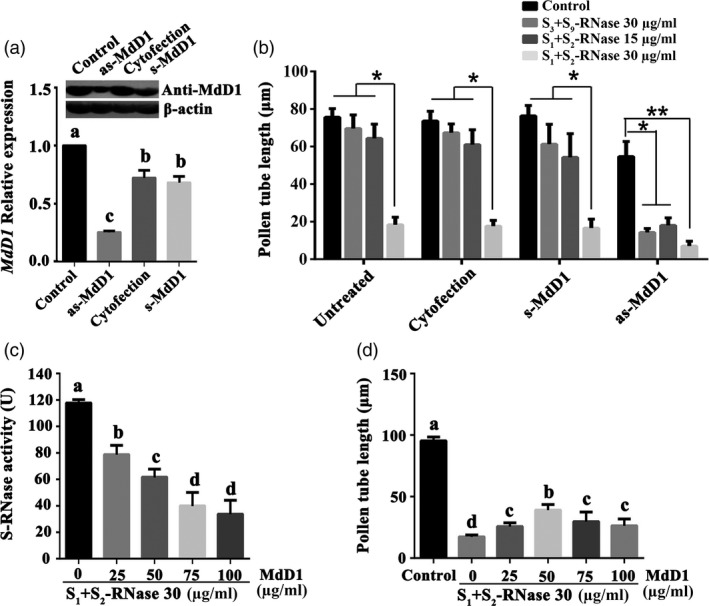
MdD1 inhibits *S*‐RNase activity**.** (a) MdD1 expression level was investigated by qRT‐PCR. The pollen tubes were treated with as‐*MdD1*, s‐*MdD1* and cytofection, respectively. as‐*MdD1* is phosphorothioated antisense oligodeoxynucleotide of *MdD1*, s‐*MdD1* is phosphorothioated sense oligodeoxynucleotide of *MdD1*, cytofection is transfection agent buffer, and control is the untreated pollen tubes. Data from three biological replicates were combined using a linear mixed‐effects model. The final data were normalized to the expression levels in untreated pollen tubes (control). (b) Pollen tube length in differently treated samples. (c) Inhibition of *S*‐RNase activity by various amounts of MdD1. Recombinant GST‐tagged MdD1 protein and His‐tagged *S*‐RNase protein were expressed in *E. coli*. S‐RNase (30 μg/mL) protein was incubated with different concentration MdD1 protein, respectively. *S*‐RNase activity was measured using torula yeast RNA as the substrate. Data are the mean ± SEM. *P <* *0.05. (d) Pollen tube length in differently treated samples. Data are the mean ± SEM. At least 60 pollen tubes were measured.

As *MdD1* could be triggered by both self and non‐self *S*‐RNase, to investigate the function of *MdD1* in pollen tubes, its expression was silenced in ‘*Ralls Janet*’ pollen tubes by using an antisense oligonucleotide assay. A significant reduction in *MdD1* transcript and corresponding protein levels were confirmed by qRT‐PCR and immunoblot analysis, respectively (Figure 4a). The growth of *MdD1*‐silenced pollen tubes was significantly inhibited by a treatment with 15 μg/mL *S*_*1*_+*S*_*2*_‐RNase, while growth was not significantly inhibited in the untransformed pollen tubes at this concentration (Figure 4b). Furthermore, we also observed that *MdD1*‐silenced pollen tube was significantly inhibited by 30 μg/mL non‐self *S*‐RNase (*S*_*3*_+*S*_*9*_‐RNase), while growth was not inhibited by the pollen tubes without silencing *MdD1* in pollen tubes at this concentration. Thus, down‐regulation of *MdD1* in pollen tubes appears to enhance the effectiveness of *S*‐RNase inhibition of pollen tube growth, suggesting that MdD1 may function as an inhibitor of RNase enzymatic activity. To test this hypothesis, purified recombinant His‐*S*‐RNase and GST‐MdD1 protein were combined in an *S*‐RNase activity assay using yeast RNA as a substrate and *S*‐RNase activity was found to decrease with increasing MdD1 concentration (Figure 4c). This trend was also seen when different recombinants *S*_*3*_+*S*_*9*_‐RNase, *S*_*2*_+*S*_*3*_‐RNase or *S*_*1*_+*S*_*9*_‐RNase were incubated with MdD1 (Figure S7). We then treated pollen tube cultures with *S*_*1*_+*S*_*2*_‐RNase and different concentrations of the MdD1 protein and found that the *S*‐RNase‐mediated inhibition of pollen tube growth was significantly reduced in the presence of MdD1 (Figure 4d). As non‐self *S*‐RNase could inhibit the *MdD1*‐silenced pollen tubes, we speculate that the MdD1 response to *S*‐RNase might occur before non‐self *S*‐RNase ubiquitination and subsequent proteasomal degradation in pollen tubes. In order to verify this hypothesis, pollen tubes were treated with the 26S proteasome inhibitor MG132, to block the 26S proteasome‐mediated ubiquitin pathway, and then added non‐self *S*‐RNase (*S*_*3*_+*S*_*9*_‐RNase). The result showed that MdD1 expression remained unchanged regardless of degradation of non‐self *S*‐RNase. This indicates that the MdD1 response to both self and non‐self *S*‐RNase in pollen tubes occurs before non‐self *S*‐RNase recognition (Figure S8).

### MdD1 interacts with *S*‐RNase

According to the phenotype of pollen tube growth which has been shown that MdD1 could inhibit *S*‐RNase activity in pollen tube, in order to investigate the mechanism of MdD1 inhibiting *S*‐RNase activity, we first investigate whether MdD1 has protease activity that could degrade *S*‐RNase directly? We purified recombinant GST‐MdD1 and His‐*S*_*1*_‐RNase, respectively. The MdD1 and *S*_*1*_‐RNase were incubated together with or without ATP, and Western blot analysis was performed to evaluate the change of *S*_*2*_‐RNase concentration. No difference was observed in the concentration between ATP‐treated and nontreated control group (Figure S9), indicating that MdD1 has no protease activity to degrade S‐RNase directly.

**Figure 5:**
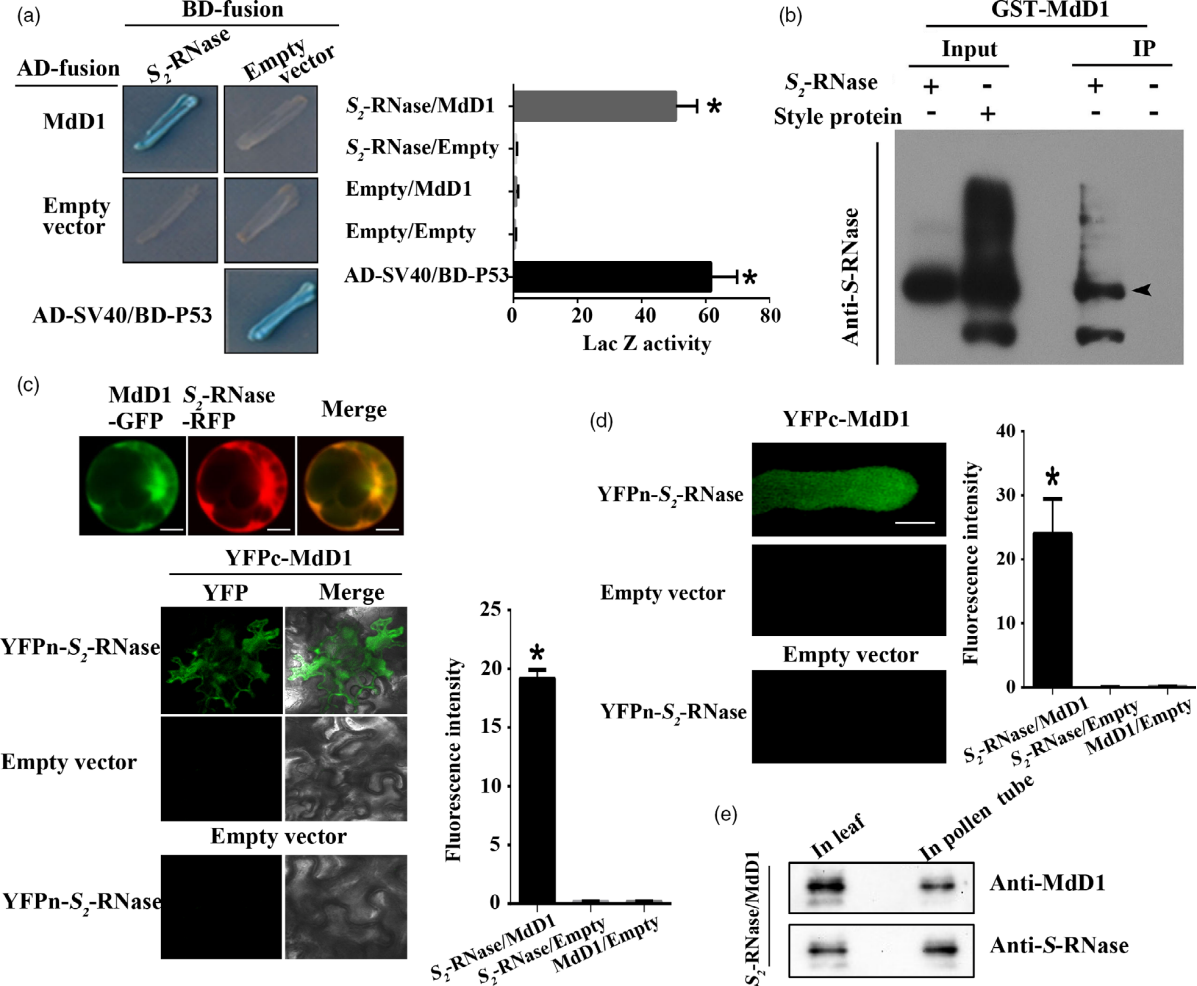
MdD1 interacts with *S*‐RNase.(a) The interactions between MdD1 and *S*‐RNase were analysed using a yeast two‐hybrid (Y2H) assay. The coding sequence of *MdD1* was ligated into the pGADT7 vector (AD, activation domain), and the coding sequence of *S*_*2*_ *‐RNase* was ligated into the pGBKT7 vector (BD, binding domain). X‐α‐gal was used as a screening marker. The *SV40* and *P53* genes were used as the positive control, and the empty AD and BD vectors as the negative control. Blue plaques indicate the interaction between two proteins. (b) A pull‐down analysis of the interaction between MdD1 and *S*_*2*_‐RNase. Purified His‐*S*_*2*_‐RNase was used as bait against purified GST‐MdD1. Bound proteins were examined using an anti‐GST antibody. GST was used as a negative control. (b) *In vitro* immunoprecipitation (IP) assay for binding between *S*_*2*_‐RNase from apple styles and the GST‐MdD1 fusion protein. The band detected by the S‐RNase antibody in the precipitated protein sample indicates the interaction between MdD1 and *S*_*2*_‐RNase. (c) Fusions of MdD1‐GFP (green fluorescent protein) and *S*_*2*_‐RNase‐RFP (red fluorescent protein) were co‐transformed into maize protoplasts. Bimolecular fluorescence complementation (BiFC) assays showed the interaction of MdD1 and *S*_*2*_‐RNase. MdD1‐YFPc and *S*_*2*_‐RNase‐YFPn were co‐injected into apple leaves. (d) BiFC assays showed the interaction of MdD1 and *S*_*2*_‐RNase in pollen tube. Scale bars = 10 μm. (e) Western blot was performed to analysis the expression of MdD1 and *S*‐RNase in pollen tube and apple leaves. These experiments were repeated three times.

As MdD1 could not function as protease, we speculated that MdD1 act as a protease inhibitor that could interact with *S*‐RNase directly. To test this hypothesis, we verified the interaction between MdD1 and *S*‐RNase. A yeast two‐hybrid (Y2H) assay was employed to determine whether an interaction occurred between MdD1 and different *S*‐RNase (*S*_*1*_‐, *S*_*2*_‐, *S*_*3*_‐ and *S*_*9*_‐RNase) haplotypes. MdD1 was found to directly interact in a non‐haplotype‐specific manner with *S*‐RNase (Figure 5a, Figure S10). A pull‐down assay using purified recombinant polyhistidine‐tagged *S*‐RNase (His‐*S*‐RNase) and glutathione S‐transferase‐tagged MdD1 (GST‐MdD1) confirmed this interaction (Figure S11). Additional evidence of interaction between MdD1 and *S*‐RNase in both pollen tube and apple leaves was obtained using bimolecular fluorescence complementation (BiFC). As shown in Figure 5c and d, fluorescence was clearly observed for the *S*‐RNase‐YFPn and MdD1‐YFPc combination. Meanwhile, as shown in Figure 5e, we performed Western bolt to test the expression of *S*‐RNase and MdD1 in pollen tube and apple leaves during the BiFC assay. Furthermore, when a competitor (*S*‐RNase) was present YFP fluorescence was weakened (Figure S12). Lastly, we performed a semi‐co‐immunoprecipitation (Co‐IP) using GST‐MdD1 purified from *E. coli* and incubated it with a total protein extract from the style of ‘*Ralls Janet*’, using an anti‐S‐RNase antibody to detect *S*‐RNase binding to MdD1. The result showed that MdD1 interacted with *S*‐RNase in the crude protein extract (Figure 5b).

### MdD1 inhibits the activity of *S*‐RNase by binding to the RNase activity sites of *S*‐RNase

**Figure 6:**
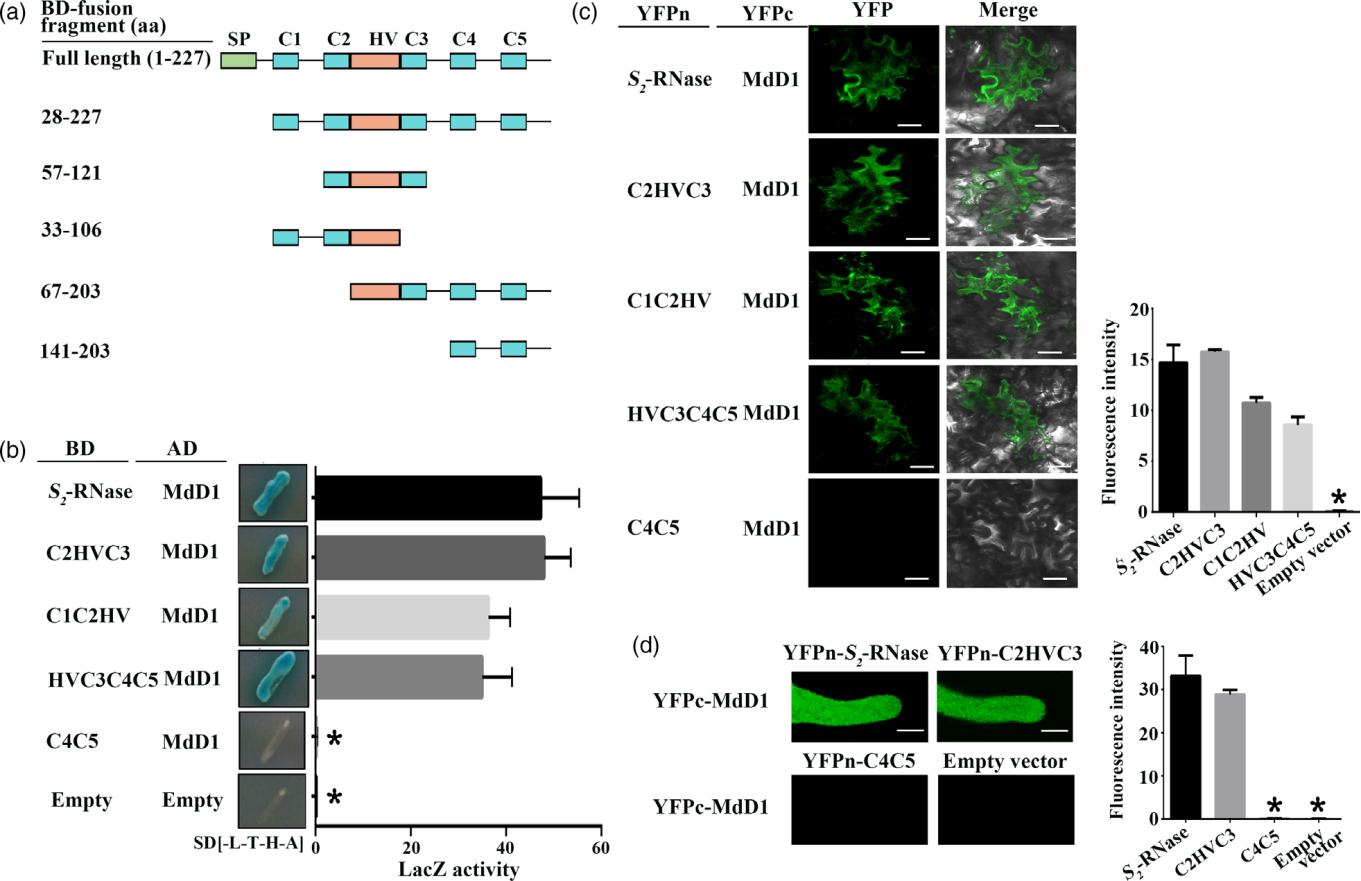
MdD1 interacts with the RNase activity site of *S*‐RNase. (a) A segment model of S‐RNase. The sequence of *S*‐RNase was divided into four fragments according to the RNase activity site of *S*‐RNase. (b) Yeast two‐hybrid (Y2H) assay showing the interactions between MdD1 and different fragments of *S*_*2*_‐RNase as described in (a). The active sites of *S*_*2*_‐RNase are localized in the C2 and C3 domains, and *S*_*2*_‐RNase was divided into four fragments. (c) Bimolecular fluorescence complementation (BiFC) assay showing the interactions between MdD1 and different fragments of the *S*_*2*_‐RNase. Scale bars = 10 μm. (d) BiFC assay showing the interactions between MdD1 and different fragments of the *S*_*2*_‐RNase in pollen tube. Scale bars = 10 μm.

As MdD1 inhibits *S*‐RNase activity and also directly interacts with *S*‐RNase, we investigated whether MdD1 inhibits *S*‐RNase activity via interaction with the active sites of *S*‐RNase. The active sites of *S*‐RNase have been identified as two histidine residues, which are located at amino acids 60 and 116 in segments C2 and C3 of the protein (Matsuura *et al*., 2001). To investigate the mechanism by which MdD1 inhibits *S*‐RNase activity, a Y2H assay was used to test the binding of each of four fragments (C1C2Hv, HvC3C4C5, C2HvC3 and C4C5) of the *S*‐RNase to MdD1 (Figure 6a). We found that MdD1 interacted with the fragments containing the active sites of *S*‐RNase (Figure 6b and Figure S13), and this was confirmed using a BiFC assay in pollen tube and apple leaves. As shown in Figure 6c and d, fluorescence was clearly observed for combinations that included MdD1‐YFPc together with *S*‐RNase‐YFPn fragments that containing the active sites. By contrast, no fluorescence was observed for the other combinations tested suggesting that MdD1 likely interacts with the active sites of *S*‐RNase *in vivo* (Figure 6c and d).

**Figure 7:**
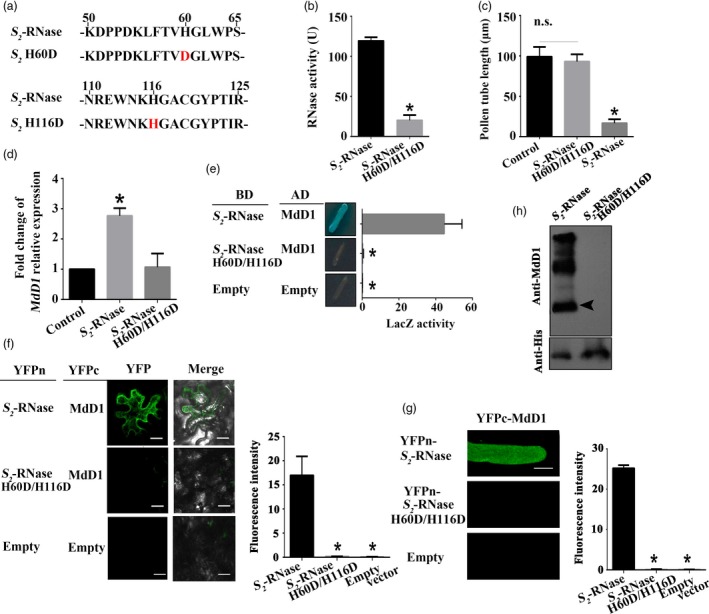
MdD1 inhibits the activity of *S*‐RNase by binding to its active sites. (a) A segment model of mutant S‐RNase. The active sites of *S*_*2*_‐RNase are two histidine (H) residues located at amino acid 60 and 116. (b) Measurement of the ribonuclease activity of *S*_*2*_‐RNase or mutated *S*_*2*_‐RNase indicated that the mutated *S*_*2*_‐RNase lost most of their ability to degrade RNA. Data are the mean ± SE. *n* = 4 for biological replicates. *, *P* < 0.05. *t*‐test. (c) Measurement of ribonuclease activity of original or mutated *S*‐RNase indicated that the mutated *S*‐RNase lost most of the ability to degrade RNA. Data are the mean values ± SEM. *P <* *0.05. (d) Measurement of pollen tube length (mutated *S*‐RNase could not inhibit pollen tube growth). Control, untreated pollen tubes; NS, no significant difference. Data are the mean ± SEM. At least 60 pollen tubes from three pistils were measured. (e) Yeast two‐hybrid (Y2H) assay showing that MdD1 interacts with the active sites of *S*_*2*_‐RNase. The active sites of *S*_*2*_‐RNase are two histidine (H) residues located at amino acid 60 and 116. Each of the histidine was mutated to an aspartic acid (D) residue, and the interactions between these *S*_*2*_‐RNases and MdD1 were investigated. The sequences corresponding to the mature peptides of *MdD1* were ligated into the pGADT7 vector (AD, activation domain), and differing mutant fragments of *S*_*2*_ *‐RNase* were ligated into the pGBKT7 vector (BD, binding domain). X‐α‐gal was used as a screening marker. The *SV40* and *P53* genes were used as the positive control, and AD and BD vectors as the negative control. Blue plaques indicate the interaction between two proteins. (f) Bimolecular fluorescence complementation (BiFC) assay showing that MdD1 was not able to interact with mutated *S*_*2*_‐RNase. MdD1‐YFPc and the various mutant fragments of *S*_*2*_‐RNase‐YFPn were co‐injected into apple leaves. Scale bars = 10 μm. (g) BiFC assay showing that MdD1 was not able to interact with mutated *S*_*2*_‐RNase in pollen tube. Scale bars = 10 μm. (h) *In vivo* immunoprecipitation (IP) assay showing that MdD1 was not able to interact with mutated *S*_*2*_‐RNase in growing pollen tubes. The band detected by the MdD1 antibody in the precipitated protein sample indicates the interaction between MdD1 and *S*_*2*_‐RNase.

To test whether MdD1 does indeed interact with the active sites of *S*‐RNase, one, or both, of the active site histidine residues was mutated to aspartic acid (D) generating lines H60D and H116D, or H60D/H116D, respectively (Figure 7a). Ribonuclease activity assays confirmed that the mutated *S*‐RNase had lost its ability to degrade RNA (Figure 7b). Furthermore, the ability of the mutated *S*‐RNase to inhibit pollen tube growth was abolished (Figure 7c). Meanwhile, we confirmed that the mutated *S*‐RNase could not trigger the up‐regulation of MdD1 in pollen tube (Figure 7d). A Y2H assay clearly demonstrated that MdD1 was not able to bind *S*‐RNase when the active sites were mutated (Figure 7e). Furthermore, a BiFC assay carried out in pollen tube and apple leaf cells also confirmed that MdD1 was unable to interact with *S*‐RNase when both active site residues were mutated, although a limited interaction was detected when only histidine 60 or histidine 116 was individually mutated (Figure 7f and g). Using a Co‐IP assay, we also observed that the MdD1 protein was immuno‐precipitated by an anti‐MdD1 antibody from extracts from the *S*‐RNase‐cultured pollen tubes, but not from extracts from the mutated *S*‐RNase‐cultured pollen tubes (Figure 7h). Taken together, these results suggest that MdD1 directly interacts with the active sites of *S*‐RNase and inhibits its activity following *S*‐RNase entry into the pollen tube.

## Discussion

Previous studies have demonstrated that SI and immunity share the same pathway (Kao and Tsukamoto, 2004; Mcclure and Franklin‐Tong, 2006, Thomas and Franklin‐Tong, 2004). SI has a lot in similarity with plant immunity, including defence against invaders. Furthermore, both SI and immunity could reject the unwanted substance or cells (Sanabria *et al*., 2008). Consistent with this notion, we found that both self and non‐self *S*‐RNase promoted the accumulation of JA in apple pollen tubes, which in turn stimulated the expression of *MdMYC2* and its target, a defensin gene, *MdD1* (Figure 3). These results imply that entry of *S*‐RNase into pollen tubes may induce a defence response pathway.

A study by Lush and Clarke showed that *Nicotiana alata* pollen tubes challenged with self *S*‐RNases are capable of recovering from inhibition if the top of the incompatible pistil is grafted onto a compatible pistil, indicating that pollen rejection may occur through a triggered mechanism, rather than a directly executed process (Lush and Clarke, 1997). Indeed, in general, when an extracellular protein enters a cell, more complex processes are initiated for it to exert its function with signal transduction or defence reactions often being involved (Sawamoto *et al*., 1996; Wang *et al*., 2009). During pollination, style‐specific determinants may trigger signalling pathways or defence responses in the pollen, suggesting similarities between defence signalling and SI (Baxter *et al*., 2014; Thomas and Franklin‐Tong, 2004; Wang *et al*., 2010; Wilkins *et al*., 2011). Several studies have shown that ROS can induce the accumulation of plant hormones (Baxter *et al*., 2014; Xu *et al*., 2015), and JA plays important roles in regulating plant defence, such as responding to ROS signalling and in regulating various developmental processes, including fertility and reproduction (Rao *et al*., 2000; Wasternack, 2007; Wasternack *et al*., 2013; Yuan and Zhang, 2015). In this study, we found that *S*‐RNase stimulated an increase in JA content (Figure 1a), suggesting that JA signalling plays a role in the pollen tube response to both self and non‐self *S*‐RNase.

MYC2 is one of the most important transcription factors in JA signalling (Kazan and John, 2013), and several studies have demonstrated that MYC family genes participate in pollen development (Figueroa and Browse, 2012; Kazan and John, 2013); nevertheless, their specific role remains unclear. Here, we found that *S*‐RNase‐induced *MdMYC2* expression is dependent on the JA signalling pathway in apple pollen tubes (Figure 1c), suggesting that a similar molecular mechanism exists in both plant defence and *S*‐RNase‐based SI in apple. Consistent with these results, a study by Shi *et al*. (2017) on *S*‐RNase‐based SI in pear found that expression of genes involved in the ‘plant hormone signal transduction pathway’ and ‘plant–pathogen interaction pathway’ was significantly enriched in self‐ and cross‐pollinations (Shi *et al*., 2017). Furthermore, previous reports have indicated that MYC2 can bind G‐box‐related motifs (Gangappa and Chattopadhyay, 2013; Kazan and John, 2013), and in this study, we identified a new signalling component, *MdD1*, using a ChIP‐seq approach (Figure 2). The G‐box promoter motif of *MdD1* was shown to bind MdMYC2 in both Y1H and ChIP‐PCR assays (Figure 3 a,b). Further, a GUS activity analysis revealed that MdMYC2 promotes the activity of the *MdD1* promoter in response to MeJA (Figure 3c).

*MdD1* is a defensin gene belonging to the γ‐thionin subfamily (Pelegrini and Franco, 2005). It is predicted to encode a secreted cysteine‐rich peptide (CRP), many of which have been reported to play roles in pollination and defence against pathogen infection (Bircheneder and Dresselhaus, 2016; Marshall *et al*., 2011). For example, in the Brassicaceae (Cruciferae), the pollen coat‐derived ligand S‐locus cysteine‐rich/S‐locus protein 11 (SCR/SP11) is a member of the defensin‐like subfamily of CRPs (Takayama *et al*., 2001). During SI, SCR/SP11, which acts as the male determinant, binds a membrane‐spanning serine/threonine receptor kinase to activate a signalling cascade, leading to the arrest of pollen tube growth (Kachroo and Nasrallah, 2002). Furthermore, *At*PCP‐Bs, also small secreted CRPs, are key regulators of the hydration ‘checkpoint’ in establishment of pollen–stigma compatibility in *Arabidopsis thaliana* (Wang *et al*., 2016). In poppy (*Papaver rhoeas*), the S‐determinant PrsS, a CRP that is secreted by the stigma, interacts with a pollen tube surface receptor S‐determinant, PrpS, inducing increased levels of Ca^2+^ and ROS (Franklin‐Tong *et al*., 2002; Wheeler *et al*., 2010). Additionally, a number of CRPs have been identified as signalling molecules, including pollen‐derived LAT52, a member of the Kunitz trypsin inhibitor‐like subfamily of CRPs that plays a role in tomato (*Solanum lycopersicum*) pollen tube germination (Tang *et al*., 2002). Another type of secreted CRP that directly binds to the tip of the growing pollen tube is the short‐range pollen tube‐attracting LURE peptides (Okuda and Higashiyama, 2010; Okuda *et al*., 2009; Takeuchi and Higashiyama, 2016). Taken together, these studies indicate that CRPs play important roles throughout the entire process of pollen tube development and growth.

Although defensins are a major class of the CRP family, there is no evidence to date that they participate in defence‐related processes in pollen tubes. *MdD1* is a defensin gene belonging to the γ‐thionin subfamily. Thionins are an important component of the plant nonspecific defence system, and they can inhibit the growth of many pathogenic microorganisms (Pelegrini and Franco, 2005). At present, most thionins isolated from plants have broad‐spectrum antifungal or bacterial characteristics (Loeza‐Ángeles *et al*., 2008). In this study, we found that MdD1 could be induced by both self and non‐self *S*‐RNase suggesting this is a defensive response mounted in both compatible and incompatible tubes (Figure 3d). This adds weight to the hypothesis that MdD1 is a component of an ancient defence system acting as a first line of defence in pollen tubes following perception of RNase. Interestingly, the silencing of *MdD1* in pollen tubes strengthened the inhibition of both self/non‐self *S*‐RNase‐treated pollen tubes (Figure 4), our data also proved that MdD1 inhibited *S*‐RNase activity by interacting with its active site (Figure 6). These results suggest that MdD1 may play a protective role during the early stage after entry of *S*‐RNase by reducing the RNase activity of *S*‐RNase in pollen tube. Furthermore, in this work, we found that there was no change of MdD1 expression between MG132‐treated and nontreated pollen tubes, indicating that MdD1 functions prior to self/non‐self recognition. Importantly, both self and non‐self *S*‐RNase has the potential to exhibit cytotoxicity via RNA degradation in pollen tubes early in the pollen–pistil interaction, and both of them are considered as a kind of invasion of exogenous substances in pollen tubes. It has been reported that the self‐recognition in SI is familiar to immunologists and the plant innate immune response is operated by a two‐phase pathogen recognition mechanism (Jones and Dangl, 2006). Additionally, both recognition and defence responses are early reaction in plant–pathogen interactions. In lichen symbionts, the recognition‐related genes were triggered only after physical contact; in the meanwhile, the defence‐related genes showed significant changes in early stage (Athukorala and Piercey‐Normore, 2015). In this study, we found that MdD1 was triggered by both self and non‐self *S*‐RNase before self/non‐self recognition. While this may be desirable in a self‐pollination, cross‐pollen tube growth would also be inhibited with the potential for activation of further downstream processes leading to death of the pollen tube via PCD. Thus, MdD1 likely acts as primary defence factor during the SI process preventing cellular damage from *S*‐RNase, which in turn would guarantee normal pollen tube growth before self/non‐self recognition. In conclusion, our study has revealed for the first time that a plant hormone‐mediated defence mechanism induced by *S*‐RNase in pollen tubes interfaces with gametophytic SI in the Rosaceae. MdD1, identified in this study as a key component of this system, acts to inhibit both self and non‐self S‐RNase activity during the initial phases of pollen tube growth in the style. This work adds a new dimension to studies on SI and makes some important links between SI and plant defence that previously have not been explored.

## Experimental procedures

### Plant materials

Pollen, styles, leaves, sepals, filaments and petals were collected from Malus domestica cv. ‘*Ralls Janet*’ (*S*_*1*_ *S*_*2*_) and stored at −80 °C until further use.

### Pollen tube growth and *in vitro* bioassay for apple SI

‘*Ralls Janet*’ pollen (*S*_*1*_ *S*_*2*_) was cultured in liquid germination medium (10% (w/v) sucrose, 0.01% (w/v) H_3_BO_3_ and 0.015% (w/v) CaCl_2_ (pH 5.8)) at 23 °C for 40 min in darkness. For the SI treatment *in vitro*, the nucleotide sequences encoding mature *S*_*1*_‐ or *S*_*2*_‐RNase peptides were amplified and ligated into the pEASY‐E1 vector (Transgene, Beijing). The transformation of the resulting plasmids into *Escherichia coli* BL21 and the recombinant His‐*S*‐RNase proteins were purified as previously described (Meng *et al*., 2014). Inactivation of purified *S*‐RNase was achieved through treatment of the sample in a boiling water bath. Primer sequences are shown in Supplemental Table S2.

### Measurement of JA in pollen tubes

‘*Ralls Janet*’ pollen was cultured in liquid germination medium for 40 min in darkness at 23 °C, the recombinant *S*‐RNase proteins were added into the medium for 20 min, and then, the pollen tubes were collected. As a control, pollen tubes were grown in germination media without recombinant *S*‐RNase proteins being present. JA levels were measured by the Zoonbio Biotechnology Company in Nan Jing using high‐performance liquid chromatography (HPLC). After *S*‐RNase treatment, the pollen tubes were ground into powder with liquid nitrogen. 1 g of pollen tube powder was collected, and 10 mL of isopropanol/hydrochloric acid was added to the powder, which was shaken at 4 °C for 30 min. After that, 20 mL of dichloromethane was added and then shaken at 4 °C for 30 min. Samples were centrifuged for 5 min at 14,000 rpm and take out the lower organic phase, while the organic phase was dried with nitrogen and dissolved with 150 μL of methanol (0.1% formic acid) in dark. After filtered with membrane of 0.22 μm in diameter, the obtained sample was detected by HPLC‐MS/MS. The standard solutions of JA with gradient of 0.1, 0.5, 1, 5, 20, 50 and 200 ng/mL were prepared with methanol (0.1% formic acid) as solvent. Chromatographic column was Agilent ZORBAX SB‐C18 reversed‐phase column (2.1 × 150, 3.5 μm). Column temperature was 30 °C. Mobile phase: A:B = (methanol/0.1% formic acid): (water/0.1% formic acid). The sample volume used for testing is 2 μL. The mass spectrometry conditions were as follows: air curtain gas was 15 psi, spray voltage was 4500 V, atomization pressure was 65 psi, auxiliary pressure was 70 psi, and atomization temperature was 400 °C.

### RNA isolation, RT‐PCR and qRT‐PCR analysis

Total RNA was extracted from pollen tubes and other tissues using the SV Total RNA Isolation System (Promega), according to the manufacturer's instructions. To eliminate genomic DNA contamination, the RNA was treated with DNase I (Takara Bio) for 20 min. First‐strand cDNA was synthesized from total RNA using an RNA PCR Kit (Takara Bio). qRT‐PCRs (20 μL volume containing 1.5 μL cDNA as the template) were performed using an ABI 7500 Real‐Time PCR System (Applied Biosystems), following the manufacturer's instructions and using SYBR Premix Ex Taq (Perfect Real Time; Takara Bio). The qRT‐PCR was conducted with three biological replicates, and each sample was analysed at least in triplicate and normalized using MdACTIN (forward primer 5′‐GTCAGTACCGTGGGAGGGTA‐3′ and reverse primer 5′‐ ACCTCTCGCATGCTAAGC‐3′) as an internal control. Transcription levels were assessed using the 2–▵▵CT method (Schmittgen and Livak, 2008). The primer sequences used for qRT‐PCR analyses are listed in Additional file 13: Table S2.

### Bioinformatics analysis

Phylogenetic analyses were performed using MEGA version 5 and the neighbour‐joining method with 1000 bootstrap replicates. The transcript factor binding motif analysis was performed using MEME (http://meme-suite.org/meme_4.11.1/).

### ChIP‐seq and ChIP‐PCR assays

The CDS region of *MdMYC2* was cloned from cDNA of pollen tubes of ‘*Ralls Janet*’. The PCR product was ligated into the pEASY‐E1 vector (Transgene, Beijing). The transformation of the resulting plasmids into Escherichia coli BL21 (DE3) and the recombinant His‐MdMYC2 proteins were purified as previously described (Meng *et al*., 2014). Primer sequences are shown in Supplemental Table S2. The MdMYC2 antibody was prepared by the ABclone Company in Wu Han. Both ChIP‐seq and ChIP‐PCR assays were completed by the ABclone Company in Wu Han. Total DNA was extracted from apple pollen tubes. The ChIP‐seq assay was performed by Illumina HiSeq 2500 sequencing platform.

### Y1H assay

The coding sequence of *MdMYC2* (2097 bp) was ligated into the pGADT7 vector. A MdD1 promoter fragment (30 bp) was ligated into the pHIS2 vector (Clontech). All constructs were transformed into yeast strain AHY187 by the lithium acetate method. The Y1H assay was conducted as described according to the manufacturer's instructions. All primers used are listed in Additional file 13: Table S2.

### GUS analysis

The *MdD1* promoter sequence (1336 bp) was ligated into the pCambia1305 vector to generate the GUS reporter construct. The coding sequence of *MdMYC2* was ligated into the pBI121 vector to generate the effector construct. Co‐transfection of the reporter and effector constructs into tobacco leaves was performed as described by Li (Li *et al*., 2016). The expression level of GUS was quantified using qRT‐PCR, and this value was used to represent GUS activity levels. The qRT‐PCR assay was described above. The PCR primers used are listed in Additional file 13: Table S2.

### Y2H assays

The Matchmaker GAL4 Two‐hybrid System (Clontech) was used for the Y2H assays. The coding sequence of MdD1 was cloned into the pGADT7 (Clontech) vector, while the coding sequences of *S*_*1*_‐, *S*_*2*_‐, *S*_*3*_‐ and *S*_*9*_‐RNase were cloned into the pGBKT7 vector. All primers used are listed in Additional file 13: Table S2. The GAL4 Y2H assay was performed according to the manufacturer's instructions. At least three independent experiments were performed.

*S*‐RNase was divided into four fragments according to its active sites. All primers used are listed in Supplemental Table S2. According to the active sites of *S*‐RNase, we then mutated one, or both, of the histidine residues of the predicted *S*‐RNase active sites to aspartic acid (D) to generate the lines H60D and H116D, or H60D/H116D, respectively. All primers used are listed in Additional file 13: Table S2.

### Pull‐down assays

The coding sequence of *MdD1* was cloned into the expression vector, pGEX4T‐1. The GST‐MdD1 fusion construct was transformed into Escherichia coli BL21 (DE3), and the recombinant protein, GST‐MdD1, was expressed and purified as previously described (Meng *et al*., 2014). The sequences corresponding to the mature peptides of *S*_1_‐, *S*_2_‐, *S*_3_‐ and *S*_9_‐RNase peptides were cloned into the pEXSY‐E1 vector (Transgene, Beijing). The PCR primers used are listed in Additional file 13: Table S2. The recombinant His‐S_1_‐RNase, His‐S_2_‐RNase, His‐S_3_‐RNase and His‐S_9_‐RNase proteins were expressed and purified as previously described (Meng *et al*., 2014).

For the pull‐down assay, the purified fusion protein, GST‐MdD1, was incubated with each of the four His‐*S*‐RNase recombinant proteins and applied to amylase resin, as previously described by Yuan (2014). Proteins were detected by Western blot with anti‐GST and anti‐His as previously described (Meng *et al*., 2014).

### BiFC assays

The full‐length MdD1 and *S*_1_‐, *S*_2_‐, *S*_3_‐ and *S*_9_‐RNase cDNA sequences were cloned into vectors harbouring a yellow fluorescent protein (YFP) coding sequence to generate either N‐terminal or C‐terminal fusions (Meng *et al*., 2014; a). The PCR primers used are listed in Supplemental Table S2. The resulting constructs were transiently expressed in apple leaves as previously described (Meng *et al*., 2014). The YFP fluorescence was imaged 5 days after transformation using an Olympus BX61 confocal laser scanning microscope. The excitation wavelength for YFP fluorescence was 488 nm, and emission fluorescence was detected at 500–542 nm.

### Transient expression of MdD1 in apple pollen and maize protoplasts

The full‐length cDNA of MdD1 was cloned in‐frame into the pEZS‐NL vector. The PCR primers used are listed in Supplemental Table S1. MdD1 was transiently expressed in ‘*Ralls Janet*’ pollen as a green fluorescent protein (GFP)‐fusion driven by the LAT52 pollen‐specific promoter. The MdD1‐GFP construct was transformed into pollen by particle bombardment, and GFP expression was visualized 2 h after transformation using fluorescence microscopy (Olympus BX61). To determine the subcellular localization of MdD1 and *S*‐RNases in maize protoplasts, the full‐length cDNAs of MdD1 and *S*_*2*_‐RNase were cloned into the pEZS‐NL vector driven by the cauliflower mosaic virus promoter (35S). The constructs 35S‐MdD1‐GFP and 35S‐S2‐RNase‐RFP (red fluorescent protein) were transiently expressed in maize protoplasts. Maize protoplast preparation and transformation were performed as previously described (Han *et al*., 2015). The transformed protoplasts were imaged for GFP/RFP fluorescence using an Olympus BX61 confocal laser scanning microscope. For excitation of the fluorescent proteins, an argon ion laser and the following wavelengths were used: 488 nm for GFP and 543 nm for RFP. Fluorescence was detected at 493–542 nm for GFP and 578–625 nm for RFP.

### Protein extraction from pollen tubes and immunoblotting

Pollen tubes were collected from liquid medium by centrifugation at 3500 g and washed three times with cold PBS buffer (0.1 m Na_2_HPO_4_ and 0.1 m NaH_2_PO_4_, pH 7.0). Pollen proteins were extracted as previously described (Rudd *et al*., 1996). Antibodies against the MdD1 recombinant proteins (anti‐MdD1) were raised in rabbits (CW Biotech). The immunoblot analyses were performed as described by Yuan (Meng *et al*., 2014).

### Immunoprecipitation assay

For the *in vitro* immunoprecipitation of MdD1 and *S*‐RNase, the recombinant protein, GST‐MdD1, was expressed and purified as previously described (Meng *et al*., 2014). GST‐MdD1 was incubated with Glutathione Sepharose 4B (GE Healthcare), but do not wash from the resin. Pistil proteins were extracted as described (Wang *et al*., 2006). The pistil crude proteins were incubated with GST‐MdD1 protein which bond to Glutathione Sepharose 4B for 2 h at 4 °C. We then washed with cold PBS buffer (0.1 mol/L Na_2_HPO_4_ and 0.1 mol/L NaH_2_PO_4_, pH 7.0) for five times in order to take out the proteins which do not bind with GST‐MdD1 protein. For immunoblot analyses, the GST‐MdD1 protein bound to the beads were resuspended in PBS buffer and separated by 12% SDS‐PAGE gels, and then transferred to nitrocellulose membranes (Bio‐Rad) using Trans‐Blot Turbo (Bio‐Rad). Proteins were detected by Western blot with anti‐S‐RNase antibody. The SDS‐PAGE and Western blot assay were described by Yuan (Meng *et al*., 2014).

### Antisense oligo experiments

An antisense oligonucleotide experiment was performed essentially as described by Moutinho *et al*. (2001). A phosphorothioated antisense oligodeoxynucleotide (as‐ODN) and its sense control (s‐ODN) were synthesized (Taihe Biotechnology Co., Ltd.) to correspond to bp 115–178 of the MdD1 open reading frame and to bp 719‐738 of the *MdMYC2* open reading frame, in order to down‐regulate expression of the genes. The antisense and sense sequences are listed in Additional file 12: Table S1. Hydrated pollen tubes were transfected as described above, and pollen tube lengths were measured 40 min after adding S‐RNase into the culture medium. For transfection, 200 μL (10 pmol) of oligonucleotides (as‐ODN or s‐ODN), 280 μL cytofectin buffer and 40 μL cytofectin were pre‐mixed and added immediately to 2 mL germination medium. As a control, cytofectin alone was used to treat the pollen.

### *S*‐RNase activity analysis

*S*‐RNase activity was measured using torula yeast RNA as the substrate. Recombinant GST‐tagged MdD1 protein and His‐tagged *S*‐RNase protein were expressed in *E. coli*. *S*‐RNase protein (30 μg/mL) was incubated with different concentration of MdD1 protein, respectively. The reaction buffer (490 mL), containing 0.1 m imidazole hydrochloric acid (HCl) (pH 7.0), 0.1 m KCl and 2 mg RNA, was incubated at 37 °C for 10 min before the addition of the enzyme solution (10 mL). After a further incubation at 37°C for 20 min, 100 mL of stop solution (25% perchloric acid, 0.75% (w/v) lanthanum acetate) was added. Following a 30‐min incubation on ice, the solution was centrifuged at 14 000 g for 5 min. The absorbance of the supernatant at 260 nm was measured with a Hitachi U‐2000 spectrophotometer. Data shown are the means ± SEM of three independent biological replicates.

### *S*‐RNase degradation analysis

Recombinant GST‐tagged MdD1 protein and His‐tagged *S*‐RNase protein were expressed in *E. coli*. *S*‐RNase protein (30 μg/mL) was incubated with equal amount of MdD1 protein in the condition with or without ATP. After 60 min of incubation, MdD1 antibody and *S*‐RNase antibody were used to detect changes in protein content.

### Accession numbers

Sequence data from this article can be found in the GenBank/NCBI data libraries under the following accession numbers: NP_001315873.1 (MdMYC2); D50837 (*S*_*1*_‐RNase); U12199 (*S*_*2*_‐RNase); U12200 (*S*_*3*_‐RNase); and U19793 (*S*_*9*_‐RNase).

## Conflict of interest

The authors declare no conflict of interest.

## Author contributions

T.L., Z.G. and D.M. designed the research; J.D. gave critical assessment of the manuscript; Z.G., Q.Y., W.L., H.Y., J.Y. and C.L. performed the experiments; Z.G., Q.Y. and Q.C. analysed the data; Z.G., J.D., D.M., T.L. and W.L. wrote the paper with input from all the other authors.

## Supporting information


**Figure S1** Pollen tube growth in control pollen, or those treated with inactive *S*‐RNase, or different concentrations of *S*_*1*_+*S*_*2*_‐RNase and *S*_*3*_+*S*_*9*_‐RNase.

**Figure S2** Sequence alignments of the deduced amino acid sequences of MdMYC2.

**Figure S3** The expression analysis of *MdMYC2*.

**Figure S4** Genome‐wide mapping of MdMYC2 performed by ChIP‐Seq**.**

**Figure S5** The expression analysis of MdD1**.**

**Figure S6** *MdD1* relative expression in pollen tube**.**

**Figure S7** MdD1 causes a decrease in *S*‐RNase activity in different *S*‐haplotype combinations.

**Figure S8** *MdD1* relative expression**.**

**Figure S9** S‐RNase could not be degraded by MdD1.

**Figure S10** Yeast two‐hybrid analysis of the physical interaction between the mature peptide of MdD1 and the mature peptides of *S*_1_‐, *S*_*3*_‐, and *S*_*9*_‐RNase.

**Figure S11** A pull‐down analysis of the interaction between MdD1 and *S*_*1*_‐, *S*_*3*_‐, *S*_*9*_‐RNase.

**Figure S12** Bimolecular fluorescence complementation (BiFC) assay showing the interactions between MdD1 and *S*_*1*_‐, *S*_*3*_‐, and *S*_*9*_‐RNase.

**Figure S13** Yeast two‐hybrid (Y2H) assay showing the interactions between MdD1 and different *S*_*1*_‐, *S*_*3*_‐, *S*_*9*_‐RNase fragments.

**Table S1 S**equences of proteins listed in Table S1.

**Table S2** Primers and oligonucleotides used in this study.

**Table S3** The candidate genes found by ChIP‐seq.
