# Supplementary figures and images for: A gamma‐thionin protein from apple, MdD1, is required for defence against S‐RNase‐induced inhibition of pollen tube prior to self/non‐self recognition
Source: Plant Biotechnol J. 2019 May 17;17(11):2184–98. doi: 10.1111/pbi.13131 (PMC6790362; doi:10.1111/pbi.13131)

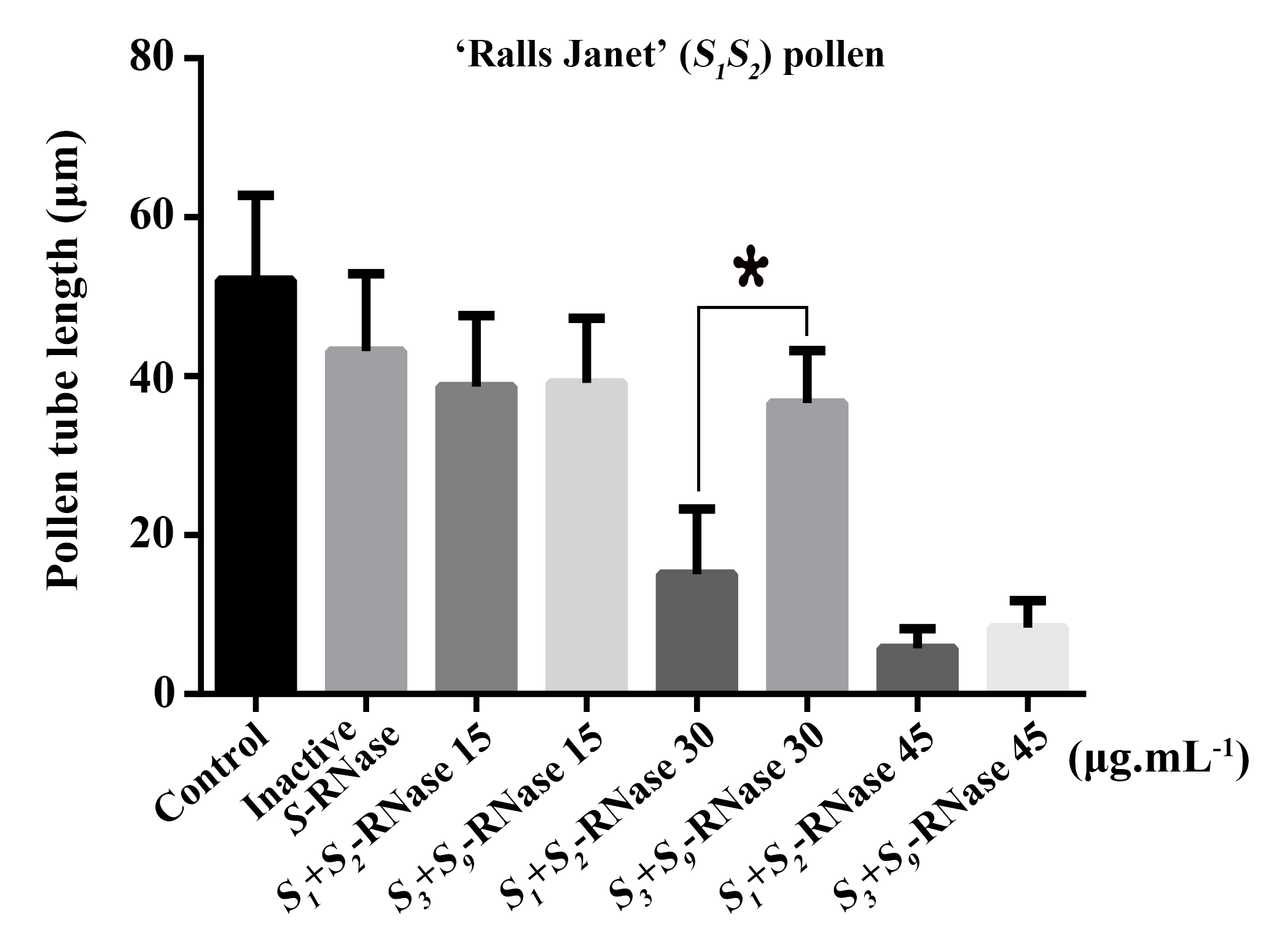

Supplement: Supplementary file 1 — Figure S1 Pollen tube growth in control pollen, or those treated with inactive S‐RNase, or different concentrations of S 1 +S 2 ‐RNase and S 3 +S 9 ‐RNase. [file PBI-17-2184-s013.tif]

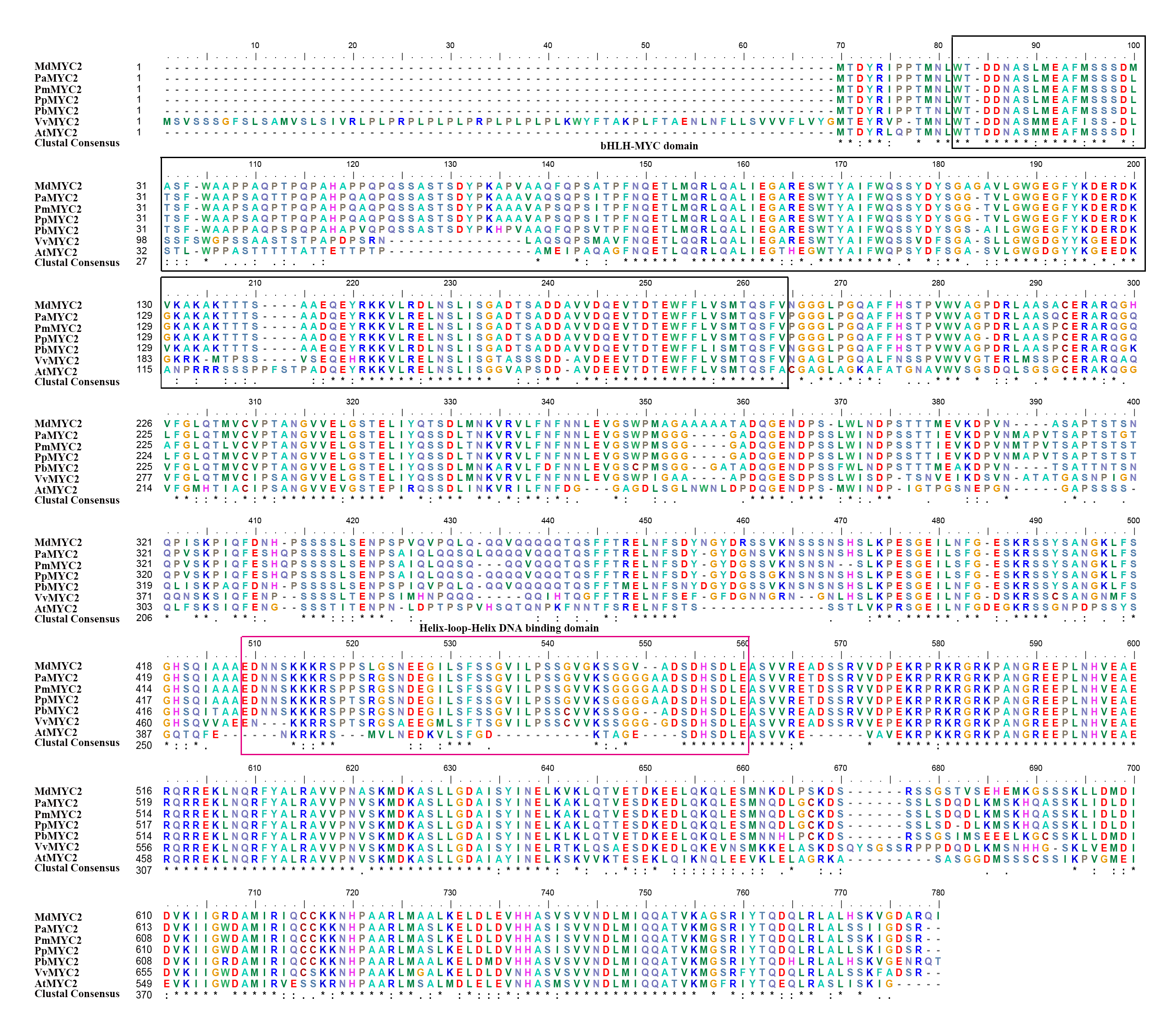

Supplement: Supplementary file 2 — Figure S2 Sequence alignments of the deduced amino acid sequences of MdMYC2. [file PBI-17-2184-s012.tif]

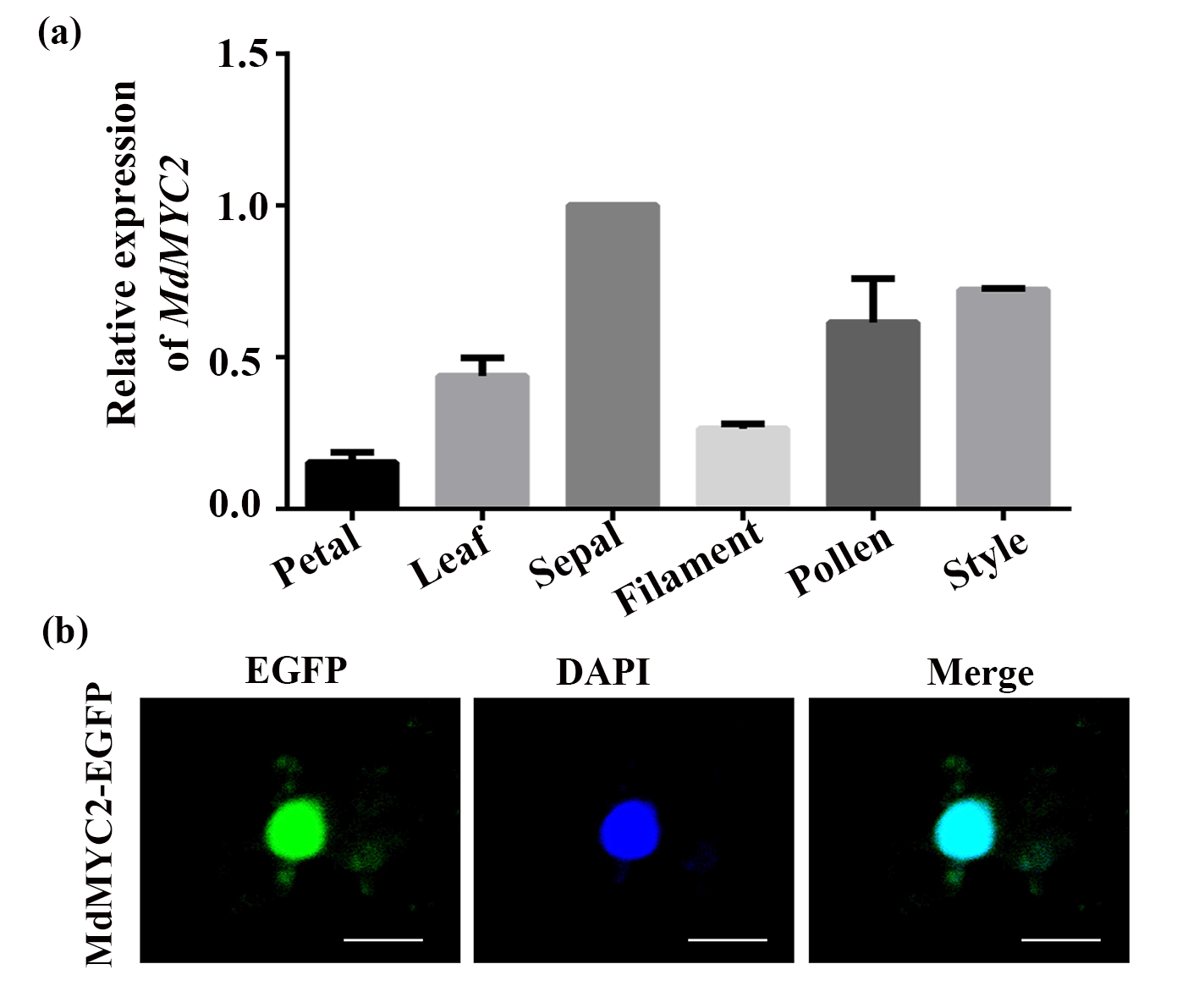

Supplement: Supplementary file 3 — Figure S3 The expression analysis of MdMYC2. [file PBI-17-2184-s011.tif]

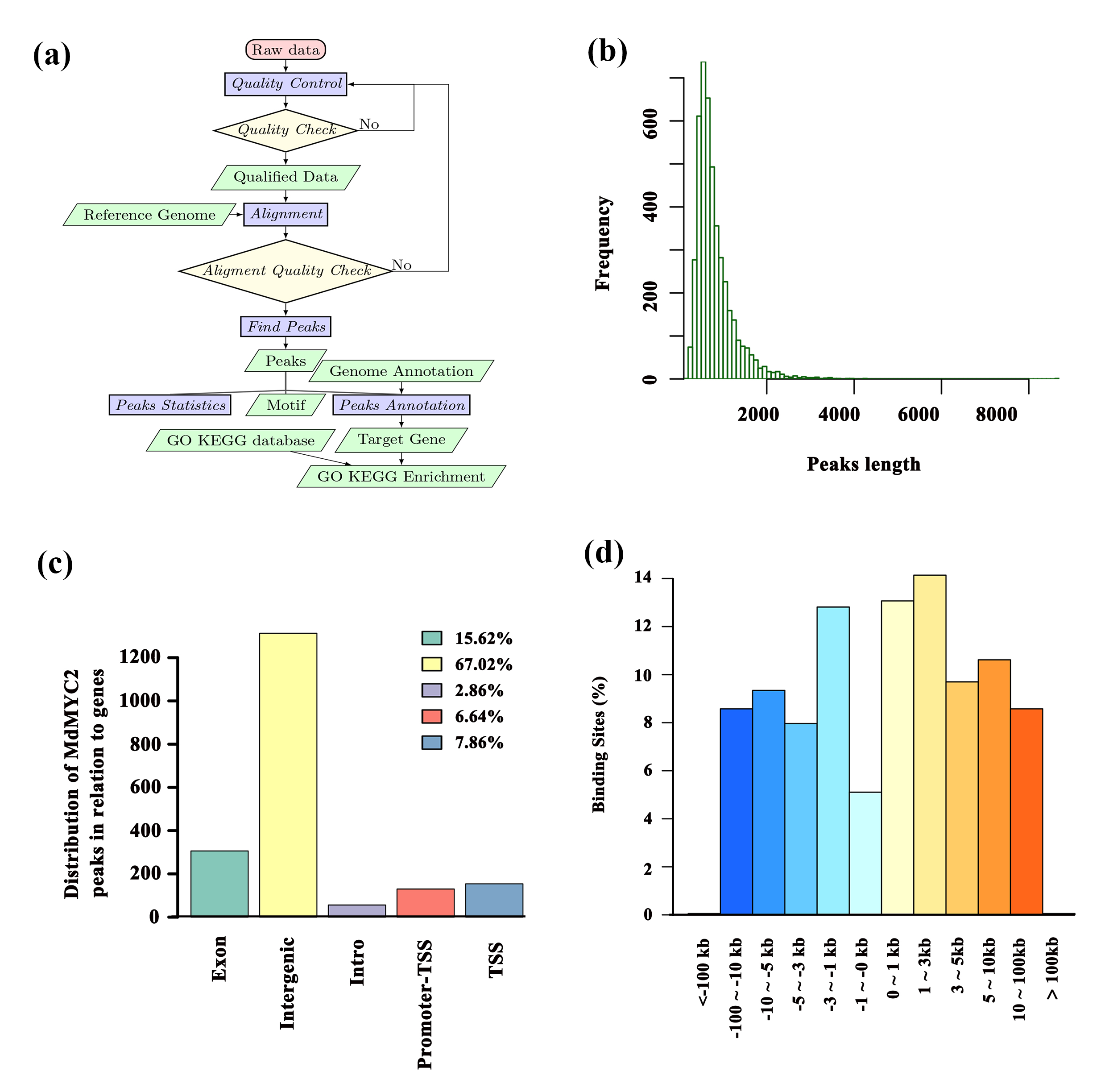

Supplement: Supplementary file 4 — Figure S4 Genome‐wide mapping of MdMYC2 performed by ChIP‐Seq. [file PBI-17-2184-s015.tif]

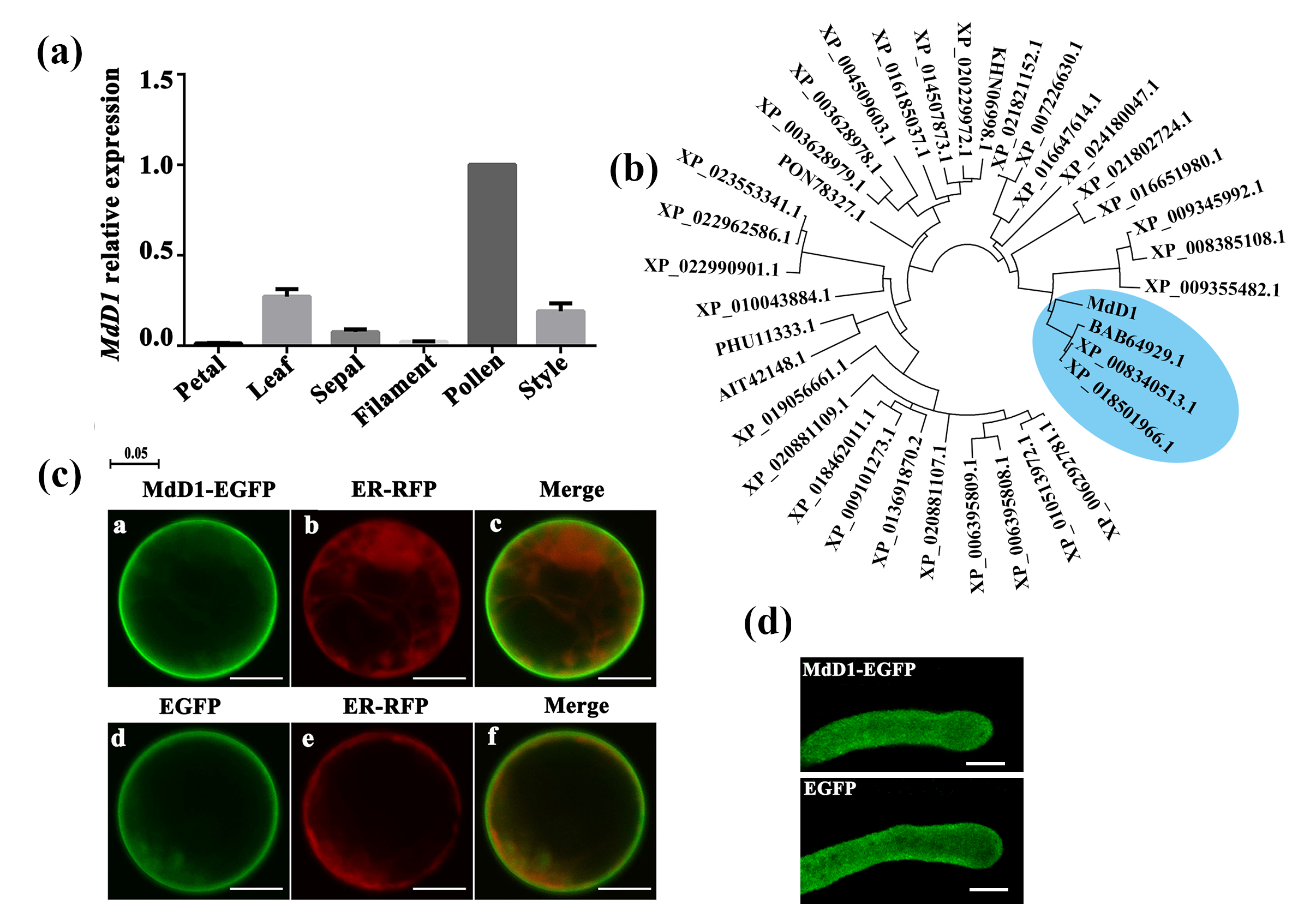

Supplement: Supplementary file 5 — Figure S5 The expression analysis of MdD1. [file PBI-17-2184-s016.tif]

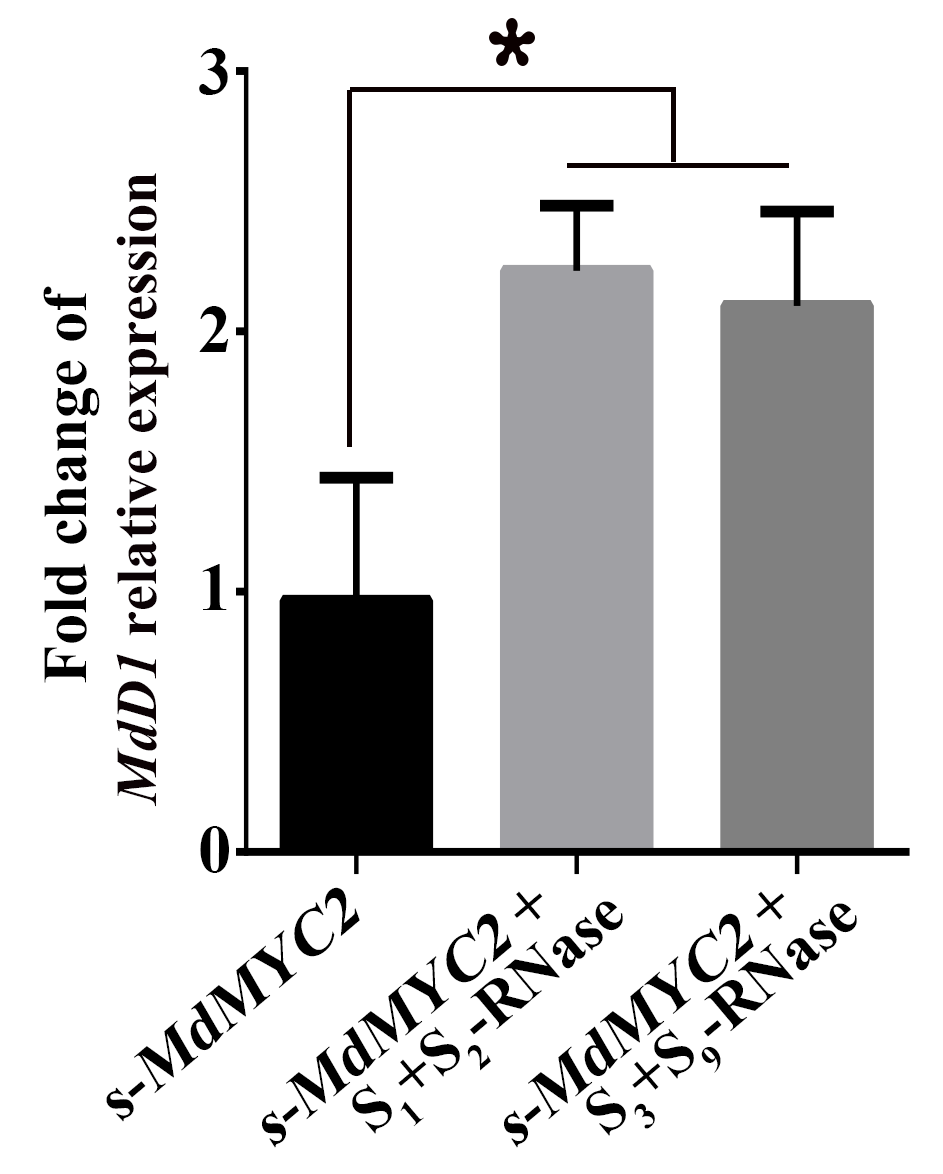

Supplement: Supplementary file 6 — Figure S6 MdD1 relative expression in pollen tube. [file PBI-17-2184-s001.tif]

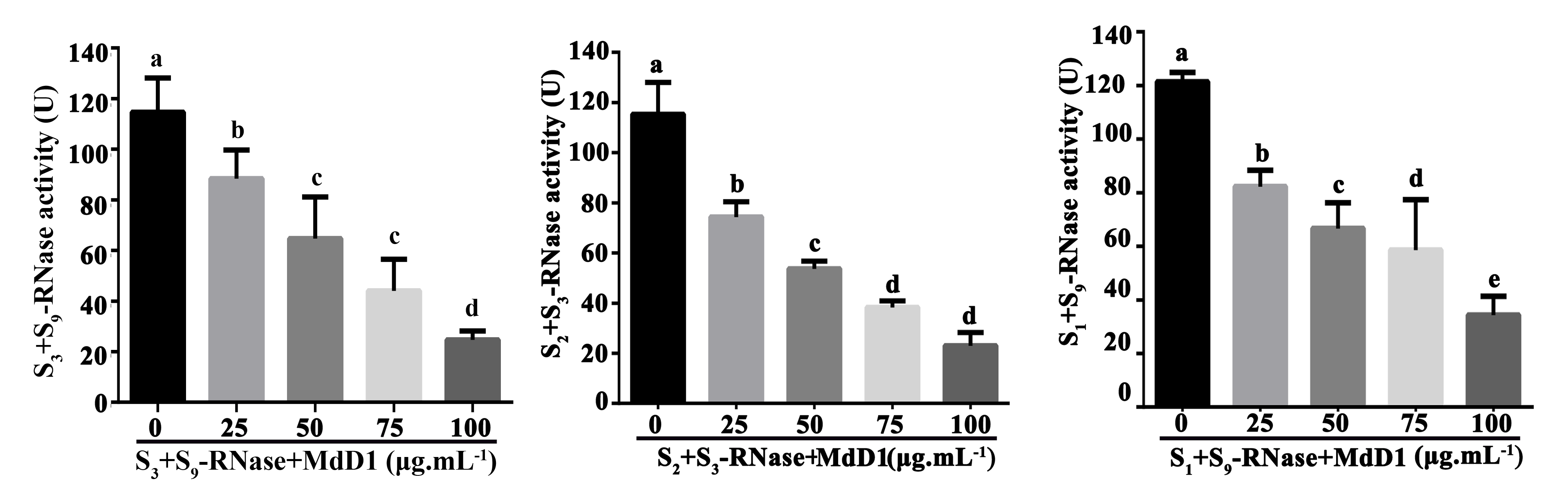

Supplement: Supplementary file 7 — Figure S7 MdD1 causes a decrease in S‐RNase activity in different S‐haplotype combinations. [file PBI-17-2184-s002.tif]

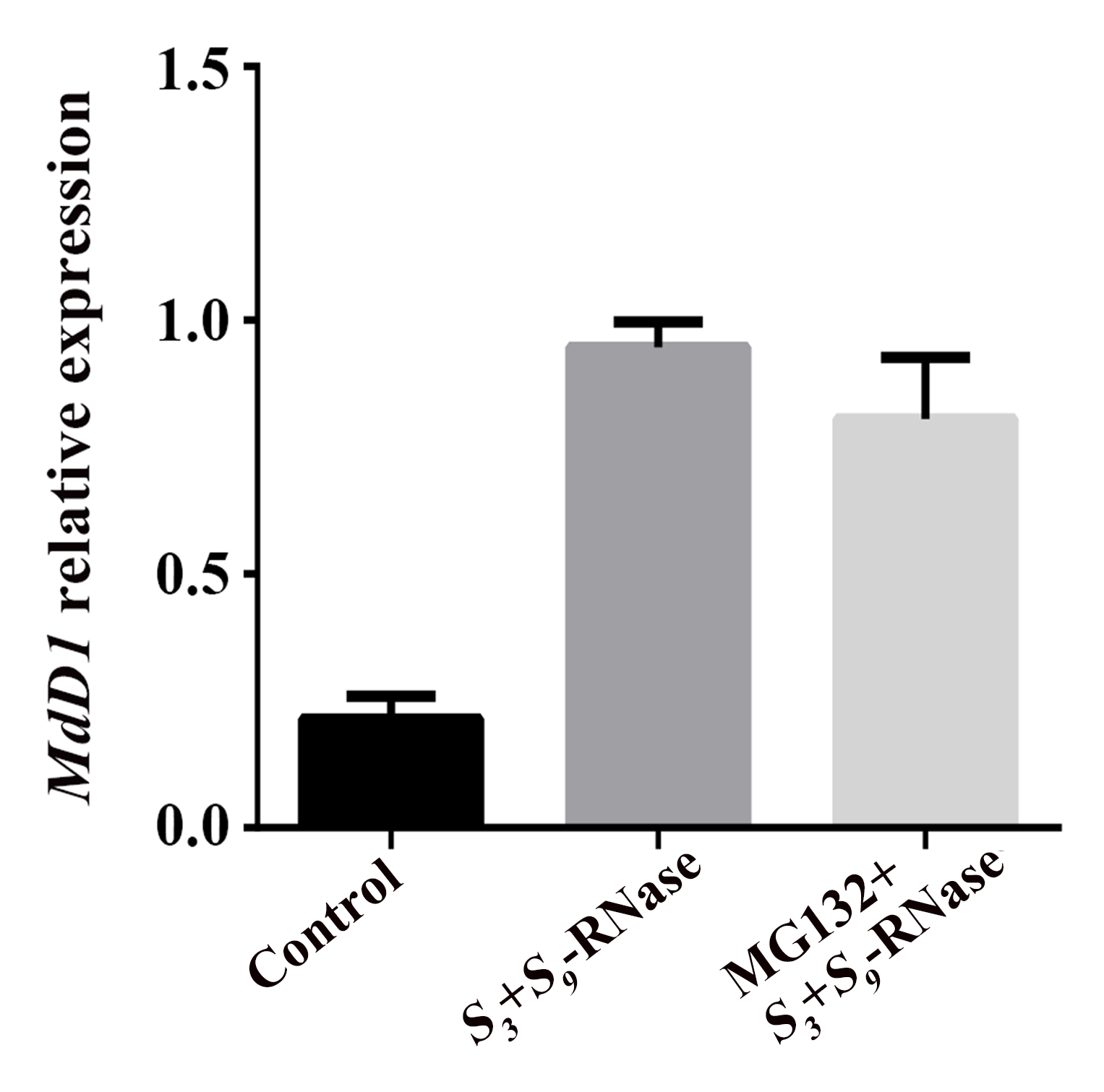

Supplement: Supplementary file 8 — Figure S8 MdD1 relative expression. [file PBI-17-2184-s003.tif]

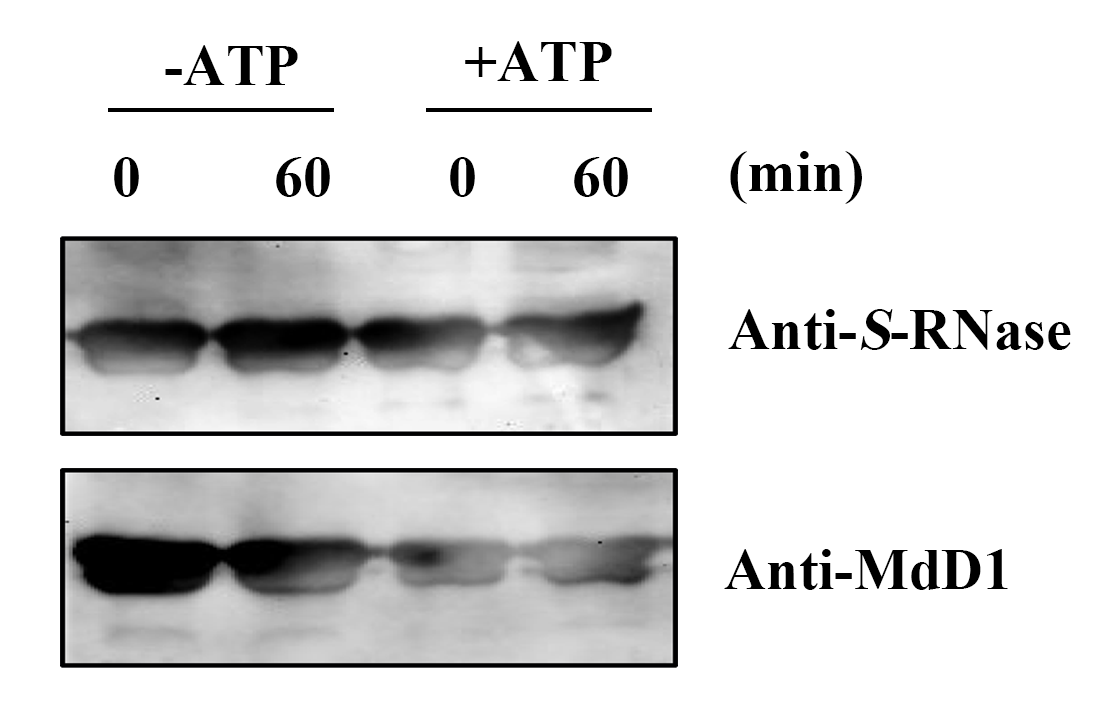

Supplement: Supplementary file 9 — Figure S9 S‐RNase could not be degraded by MdD1. [file PBI-17-2184-s004.tif]

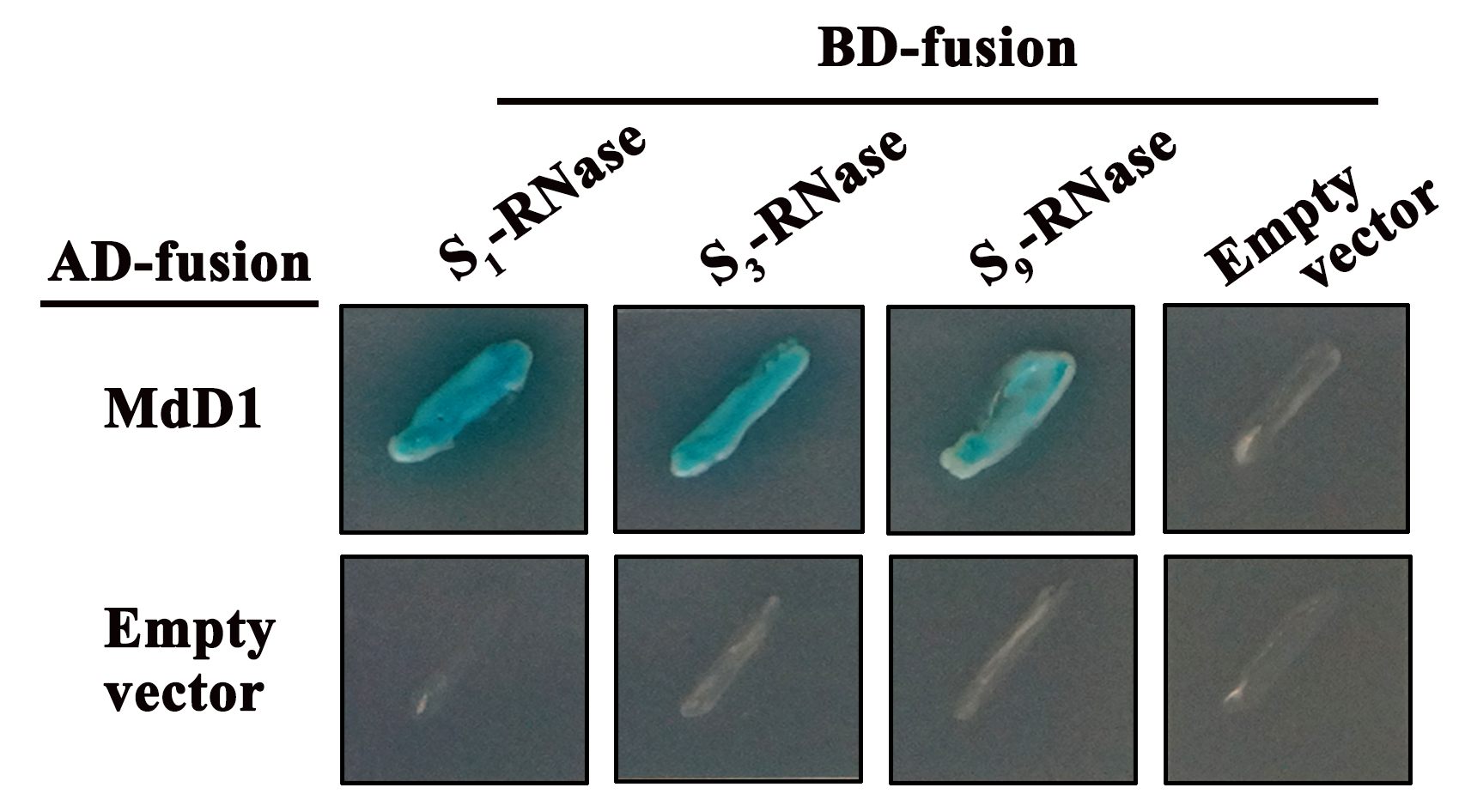

Supplement: Supplementary file 10 — Figure S10 Yeast two‐hybrid analysis of the physical interaction between the mature peptide of MdD1 and the mature peptides of S 1‐, S 3 ‐, and S 9 ‐RNase. [file PBI-17-2184-s005.tif]

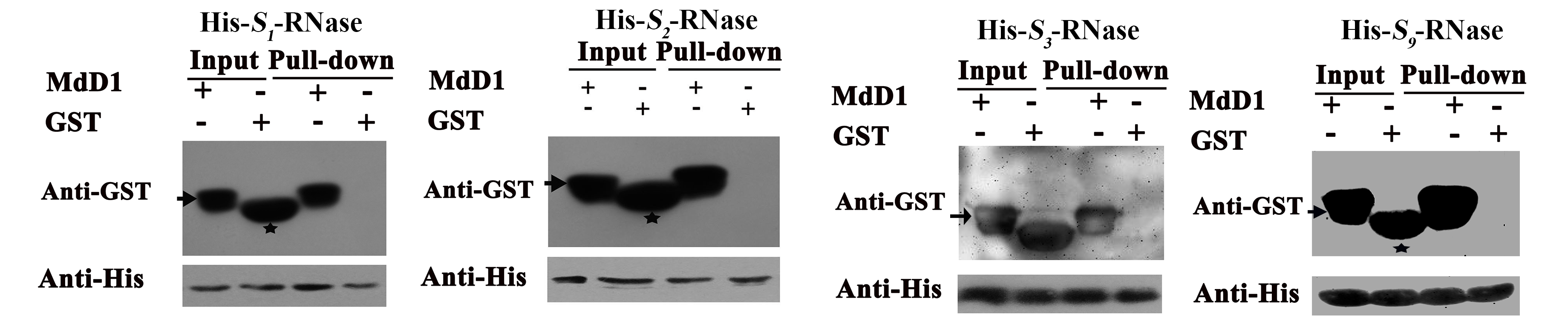

Supplement: Supplementary file 11 — Figure S11 A pull‐down analysis of the interaction between MdD1 and S 1 ‐, S 3 ‐, S 9 ‐RNase. [file PBI-17-2184-s006.tif]

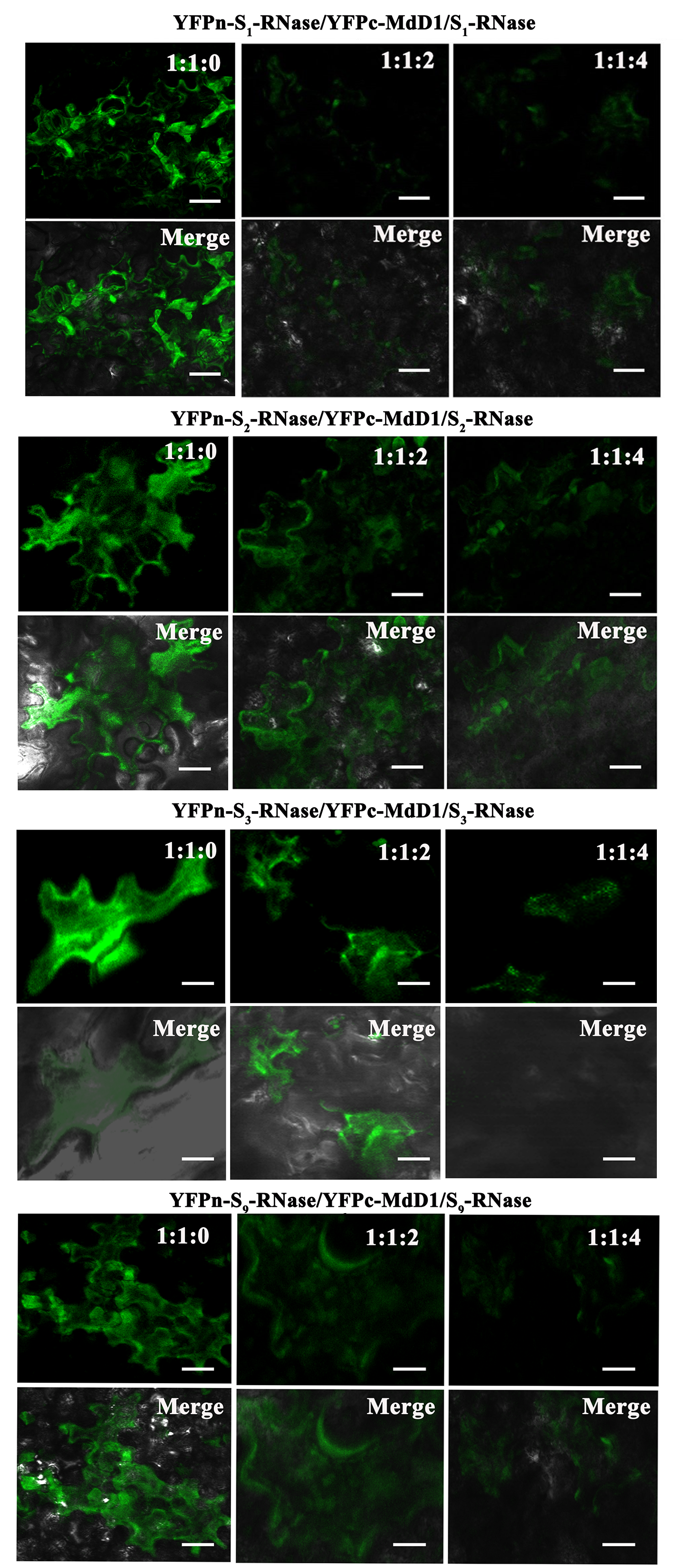

Supplement: Supplementary file 12 — Figure S12 Bimolecular fluorescence complementation (BiFC) assay showing the interactions between MdD1 and S 1 ‐, S 3 ‐, and S 9 ‐RNase. [file PBI-17-2184-s007.tif]

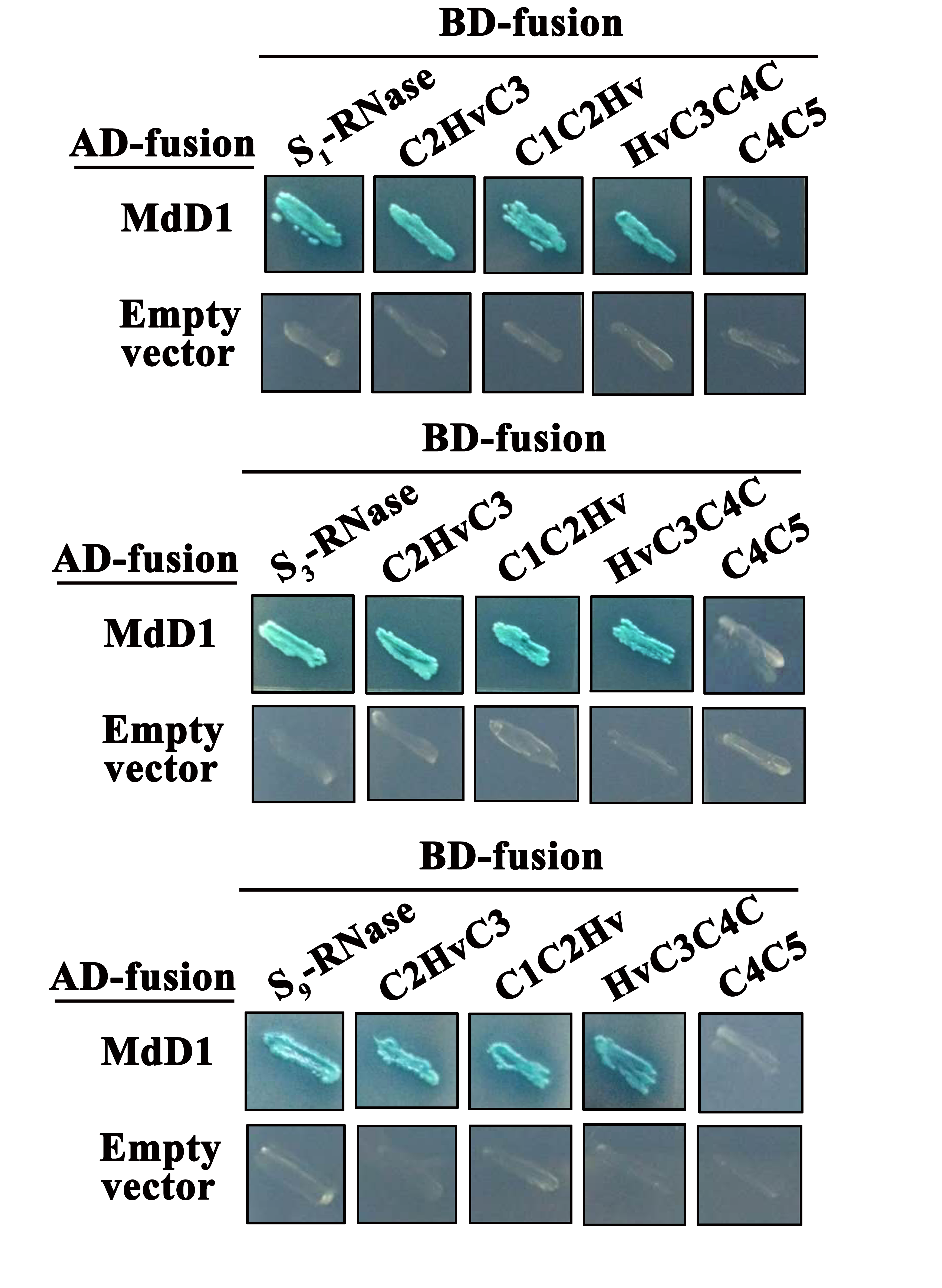

Supplement: Supplementary file 13 — Figure S13 Yeast two‐hybrid (Y2H) assay showing the interactions between MdD1 and different S 1 ‐, S 3 ‐, S 9 ‐RNase fragments. [file PBI-17-2184-s008.tif]
