# Supplementary material for: A gamma‐thionin protein from apple, MdD1, is required for defence against S‐RNase‐induced inhibition of pollen tube prior to self/non‐self recognition
Source: Plant Biotechnol J. 2019 May 17;17(11):2184–98. doi: 10.1111/pbi.13131 (PMC6790362; doi:10.1111/pbi.13131)
Supplement: Supplementary file 14 — Table S1 Sequences of proteins listed in Table S1. [file PBI-17-2184-s009.docx]

**MdD1:** MEHSMRLVSAAFVLVLLFAATEMGPMGVEARSKSDKVAKERTCEAASGKFKGLCFSSTNCKNTCKVEKFTGGQCQGFRRRCMCNKKC

**MdMYC2:**

MTDYRIPPTMNLWTDDNASLMEAFMSSSDMASFWAAPPAQPTPQPAHAPPQPQSSASTSDYPKAPVAAQFQPSATPFNQETLMQRLQALIEGARESWTYAIFWQSSYDYSGAGAVLGWGEGFYKDERDKVKAKAKTTTSAAEQEYRKKVLRDLNSLISGADTSADDAVVDQEVTDTEWFFLVSMTQSFVNGGGLPGQAFFHSTPVWVAGPDRLAASACERARQGHVFGLQTMVCVPTANGVVELGSTELIYQTSDLMNKVRVLFNFNNLEVGSWPMAGAAAAATADQGENDPSLWLNDPSTTTMEVKDPVNASAPTSTSNQPISKPIQFDNHPSSSSLSENPSPVQVPQLQQQVQQQQQTQSFFTRELNFSDYNGYDRSSVKNSSSNSHSLKPESGEILNFGESKRSSYSANGKLFSGHSQIAAAEDNNSKKKRSPPSLGSNEEGILSFSSGVILPSSGVGKSSGVADSDHSDLEASVVREADSSRVVDPEKRPRKRGRKPANGREEPLNHVEAERQRREKLNQRFYALRAVVPNASKMDKASLLGDAISYINELKVKLQTVETDKEELQKQLESMNKDLPSKDSRSSGSTVSEHEMKGSSSKLLDMDIDVKIIGRDAMIRIQCCKKNHPAARLMAALKELDLEVHHASVSVVNDLMIQQATVKAGSRIYTQDQLRLALHSKVGDARQI
