# Supplementary material for: A gamma‐thionin protein from apple, MdD1, is required for defence against S‐RNase‐induced inhibition of pollen tube prior to self/non‐self recognition
Source: Plant Biotechnol J. 2019 May 17;17(11):2184–98. doi: 10.1111/pbi.13131 (PMC6790362; doi:10.1111/pbi.13131)
Supplement: Supplementary file 15 — Table S2 Primers and oligonucleotides used in this study. [file PBI-17-2184-s010.docx]

| **Supplementary Table 2 \| Primers and oligonucleotides used in this Study.** | | |  |  |
| --- | --- | --- | --- | --- |
| Name | | Sequence (5’—3’) | Use in this study | Remark |
| GST-MdD1 | F | GAATTCATGGGACCAATGGGTGTTG | Cloning MdD1 from apple/ vector construction for GST-tagged recombinant MdD1. | The CDS of MD1 was introduced into pGEX4T-1 vector by *EcoRⅠ* and *XhoⅠ* restriction site. |
|  | R | CTCGAGTTAGCACTTTTTGTTGCAC |  |  |
| S_1_-H59D/S_2_- H60D | F | AAGTTGTTTACSGTTGAYGGTTTGTGGCCTT | For the construct of His- catalytic site mutated S1- and S2-RNase. |  |
| S_1_-H59D/S_2_- H60D | R | AAGGCCACAAACCRTCAACSGTAAACAACTT |  |  |
| AD-MdD1-Mat | F | GAATTCATGGGACCAATGGGTGTTG | Y2H assay. |  |
|  | R | CTCGAGTTAGCACTTTTTGTTGCAC |  |  |
| BK-S_1_,S_2_,S_3_,S_9_-Mat | F | GAATTCATGGATTATTWTCAATTTACGC |  |  |
| BK-S_1_-Mat | R | CTGCAGATACTGAATATTGGTGGGGCAG |  |  |
| BK-S_2_-Mat | R | CTGCAGATACAGAATATGATTGGTGGGG |  |  |
| BK-S_3_-Mat | R | CTGCAGATACTGAATATTATTGGTGGGGC |  |  |
| BK-S_9_-Mat | R | CTGCAGATACAGAATATTATTGGTGGGGC |  |  |
| qRT-MdD1 | F | GGAGCATTCTATGCGTCTTGT | qRT-PCR analysis. |  |
|  | R | CATTGGTCCCATCTCGGTAG |  |  |
| qRT-MdMYC2 | F | TTTGGGCTGCAGACTATGG |  |  |
|  | R | CGAGCCCAACTCCACAAC |  |  |
| Actin | F | GGCTGGATTTGCTGGTGATG |  |  |
|  | R | TGCTCACTATGCCGTGCTCA |  |  |
| YFPn-MdD1 | F | GGTACCATGGAGCATTCTATGCGTC | BiFC assay. |  |
|  | R | GAATTCTTAGCACTTTTTGTTGCAC |  |  |
| *S*_1_-RNase-YFPc | F | GGTACCATGGTGACGGGGATG |  |  |
|  | R | GAATTCATACTGAATATTGGTGGG |  |  |
| *S*_2_-RNase-YFPc | F | GGTACCATGGGGACTACGCGGA |  |  |
|  | R | GAATTCATACAGAATATGATTGGTG |  |  |
| *S*_3_-RNase-YFPc | F | GGTACCGGGATTACAGGGA |  |  |
|  | R | GAATTCATACAGAATATTATTGGTGG |  |  |
| *S*_9_-RNase-YFPc | F | GGATCCATGGGGATTACGGGGA |  |  |
|  | R | GTCGACATACAGAATATTATTGGTG |  |  |
| His-S_1_,S_2_,S_3_,S_9_-RNase | F | ATGGATTATTWTCAATTTACGC | Vector construction for His-tagged recombinant S-RNase and Pull down assay. | The CDS of S_1_, S_2_, S_3_, and S_9_-RNase was introduced into pEASY-E1 vector by T-A clone without any restriction site. |
| His-S_1_-RNase | R | ATACTGAATATTGGTGGGGCAG |  |  |
| His-S_2_-RNase | R | ATACAGAATATGATTGGTGGGG |  |  |
| His-S_3_-RNase | R | ATACTGAATATTATTGGTGGGGC |  |  |
| His-S_9_-RNase | R | ATACAGAATATTATTGGTGGGGC |  |  |
| pEZS-NL-MdD1 | F | CTCGAGATGGAGCATTCTATGCGTCTT | Subcellular localization assay. | The CDS of MdD1 was introduced into pEZS-NL vector by *XhoⅠ* and *BamHⅠ* restriction site. |
| pEZS-NL-MdD1 | R | GGATCCTTGCACTTTTTGTTGCACAT |  |  |
| as-ODN-MdD1 | A | CATTGGTCCCATCTCGGTAG | Antisense interference of MdD1 mRNA in apple pollen tubes. | Phosphorothioate modifications in both the 5´ and 3’ terminus (three or four bases modified in each end), indicated in lowercase. |
| s-ODN-MdD1 | S | GGAGCATTCTATGCGTCTTGT |  |  |
| as-ODN-MdMYC2 | A | CGAGCCCAACTCCACAAC | Antisense interference of MdMYC2 mRNA in apple pollen tubes. |  |
| s-ODN- MdMYC2 | S | TTTGGGCTGCAGACTATGG |  |  |
