# Supplementary material for: A gamma‐thionin protein from apple, MdD1, is required for defence against S‐RNase‐induced inhibition of pollen tube prior to self/non‐self recognition
Source: Plant Biotechnol J. 2019 May 17;17(11):2184–98. doi: 10.1111/pbi.13131 (PMC6790362; doi:10.1111/pbi.13131)
Supplement: Supplementary file 16 — Table S3 The candidate genes found by ChIP‐seq. [file PBI-17-2184-s014.docx]

**Supplementary Table 3 | The candidate genes by ChIP-seq.**

| **Plaza number** | **Peak score** | **Chr.** | **Distance.to.TSS** | **Predict function** | **Expressed tissues** |
| --- | --- | --- | --- | --- | --- |
| MD00G002820 | 51.76 | MDC000117.362 | 94 | NA | Leaf |
| MD00G104240 | 65.96 | MDC004448.135 | -461 | NA | Pollen, Leaf |
| MD00G108150 | 55.14 | MDC004619.202 | -479 | NA | Style, Leaf |
| MD00G013530 | 53.14 | MDC000558.226 | -405 | transmembrane transport;  substrate-specific transmembrane transporter activity;  integral to membrane | Leaf |
| MD00G144430 | 55.72 | MDC006197.428 | -193 | NA | Pollen, Leaf |
| MD00G014490 | 50.58 | MDC000606.34 | -606 | NA | Style, Leaf |
| MD00G148740 | 50.48 | MDC006401.143 | -535 | NA | Leaf |
| MD00G149390 | 80.28 | MDC006427.312 | -974 | NA | Leaf |
| MD00G149500 | 52.34 | MDC006432.350 | -56 | NA | Pollen, Leaf |
| MD00G171290 | 372.47 | MDC007329.509 | -67 | NA | Leaf |
| MD00G174340 | 86.11 | MDC007463.318 | -814 | Photosynthesis;  photosystem II stabilization;  photosystem II reaction center | Style, Leaf |
| MD00G181480 | 52.85 | MDC007787.396 | -668 | acid phosphatase activity; metal ion binding; cell wall | Leaf |
| MD00G183750 | 56.05 | MDC007893.240 | 41 | NA | Style, Leaf |
| MD00G191180 | 74.95 | MDC008206.581 | -716 | NA | Leaf |
| MD00G195540 | 53.86 | MDC008398.404 | -753 | NA | Leaf |
| MD00G197370 | 64.84 | MDC008469.455 | -202 | protein self-association | Pollen, Leaf |
| MD00G199560 | 52.59 | MDC008575.350 | -130 | NA | Leaf |
| MD00G220640 | 62.68 | MDC009548.346 | -232 | NA | Style, Leaf |
| MD00G224540 | 54.04 | MDC009713.269 | -661 | NA | Leaf |
| MD00G225910 | 53.35 | MDC009776.200 | -653 | NA | Leaf |
| MD00G246220 | 85.13 | MDC010601.588 | 69 | NA | Leaf |
| MD00G257830 | 54.67 | MDC011057.161 | -117 | NA | Leaf |
| MD00G027570 | 83.32 | MDC001172.233 | -299 | NA | Leaf |
| MD00G285420 | 73.51 | MDC012196.335 | -397 | NA | Leaf |
| MD00G300830 | 50.16 | MDC012799.173 | -208 | NA | Pollen, Leaf |
| MD00G302400 | 59.18 | MDC012846.95 | -852 | NA | Leaf |
| MD00G309780 | 56.15 | MDC013157.443 | -897 | NA | Leaf |
| **Plaza number** | **Peak score** | **Chr.** | **Distance.to.TSS** | **Predict function** | **Expressed tissues** |
| MD00G030160 | 50.9 | MDC001291.363 |  | regulation of transcription, DNA-dependent; sequence-specific DNA binding transcription factor activity; DNA binding | Leaf |
| MD00G320910 | 84.84 | MDC013602.229 | -969 | NA | Style, Leaf |
| MD00G322140 | 57.76 | MDC013655.199 | -330 | NA | Leaf |
| MD00G334600 | 69.34 | MDC014166.311 | -491 | NA | Pollen, Leaf |
| MD00G347230 | 243.68 | MDC015543.142 | -165 | NA | Pollen, Leaf |
| MD00G348350 | 88.91 | MDC015602.348 | -579 | NA | Leaf |
| MD00G036450 | 61.02 | MDC001545.361 | -663 | NA | Style, Leaf |
| MD00G370380 | 50.54 | MDC016751.279 | -848 | NA | Leaf |
| MD00G376540 | 71.63 | MDC017059.194 | -400 | NA | Leaf |
| MD00G379010 | 299.87 | MDC017159.85 | -93 | NA | Pollen, Leaf |
| MD00G390220 | 74.6 | MDC017677.287 | -910 | NA | Pollen, Leaf |
| MD00G039300 | 90.94 | MDC001667.242 | -879 | NA | Pollen, Leaf |
| MD00G399570 | 62.03 | MDC018183.299 | -972 | NA | Leaf |
| MD00G401380 | 92.69 | MDC018252.142 | -354 | iron-sulfur cluster binding；oxidation-reduction process；NADH dehydrogenase (ubiquinone) activity；  oxidation-reduction process | Leaf |
| MD00G412830 | 57.97 | MDC018807.211 | -156 | NA | Leaf |
| MD00G417100 | 50.71 | MDC019042.196 | 32 | NA | Pollen, Leaf |
| MD00G420370 | 72.81 | MDC019179.172 | 79 | NA | Pollen, Leaf |
| MD00G423710 | 75.35 | MDC019349.61 | -181 | NA | Style, Leaf |
| MD00G446930 | 52.35 | MDC020499.184 | 52 | NA | Leaf |
| MD00G462200 | 50.04 | MDC021386.268 | -954 | NA | Leaf |
| MD00G476160 | 52.08 | MDC022163.222 | -873 | NA | Style, Leaf |
| MD00G481590 | 51.17 | MDC022486.122 | -343 | NA | Leaf |
| MD00G485520 | 52.35 | MDC022738.115 | -806 | NA | Pollen, Style, Leaf |
| MD00G490010 | 66.53 | MDC023281.32 | -125 | NA | Leaf |
| MD00G498210 | 57.14 | MDC025870.27 | -699 | NA | Pollen, Leaf |
| MD00G514730 | 57.54 | MDC039031.3 | 22 | NA | Style, Leaf |
| MD00G040950 | 87.21 | MDC001747.422 | -169 | defense response | Pollen, Leaf |
| MD00G055320 | 72.08 | MDC002301.347 | -286 | NA | Pollen, Leaf |
| **Plaza number** | **Peak score** | **Chr.** | **Distance.to.TSS** | **Predict function** | **Expressed tissues** |
| MD00G062950 | 54.65 | MDC002624.161 | 66 | regulation of photoperiodism, flowering | Leaf |
| MD00G072610 | 54.72 | MDC003015.193 | -746 | NA | Pollen, Leaf |
| MD00G077010 | 67.77 | MDC003235.299 | 16 | NA | Leaf |
| MD00G091120 | 59.25 | MDC003897.176 | -631 | NA | Leaf |
| MD00G093980 | 52.68 | MDC004016.612 | -642 | NA | Pollen, Leaf |
| MD00G095680 | 61.35 | MDC004079.463 | -868 | NA | Leaf |
| MD10G011100 | 97.31 | 10 | -254 | NA | Leaf |
| MD10G011110 | 54.06 | 10 | -92 | NA | Leaf |
| MD10G012610 | 172.43 | 10 | 99 | proton-transporting ATPase activity, rotational mechanism;  proton-transporting ATP synthase activity, rotational mechanism;  hydrogen ion transmembrane transporter activity | Leaf |
| MD10G025780 | 51.26 | 10 | -370 | NA | Pollen, Leaf |
| MD11G014060 | 75.97 | 11 | -509 | NA | Leaf |
| MD11G014820 | 80.47 | 11 | -727 | NA | Leaf |
| MD11G015050 | 68.3 | 11 | -402 | NA | Pollen, Leaf |
| MD11G018400 | 54.43 | 11 | -604 | NA | Leaf |
| MD11G024490 | 71.94 | 11 | -253 | NA | Leaf |
| MD11G026090 | 199.48 | 11 | -802 | NA | Leaf |
| MD12G002360 | 63.78 | 12 | -518 | NA | Style, Leaf |
| MD12G005620 | 83.21 | 12 | -142 | NA | Pollen, Leaf |
| MD12G008010 | 55.26 | 12 | -869 | NA | Leaf |
| MD12G012800 | 59.05 | 12 | -722 | NA | Leaf |
| MD12G018540 | 69.4 | 12 | 548 | NA | Pollen, Leaf |
| MD12G023230 | 64.68 | 12 | -909 | NA | Leaf |
| MD13G001170 | 123.67 | 13 | -969 | regulation of transcription, DNA-dependent; phosphorelay signal transduction system; cytokinin-activated signaling pathway | Leaf |
| MD13G006010 | 52.08 | 13 | 54 | NA | Leaf |
| MD13G008800 | 75.44 | 13 | -91 | NA | Leaf |
| **Plaza number** | **Peak score** | **Chr.** | **Distance.to.TSS** | **Predict function** | **Expressed tissues** |
| MD13G024790 | 53.2 | 13 | -457 | NA | Pollen, Leaf |
| MD13G028780 | 55.68 | 13 | -36 | NA | Leaf |
| MD14G009390 | 69.98 | 14 | -92 | NA | Leaf |
| MD14G009410 | 86.29 | 14 | -984 | NA | Leaf |
| MD14G012570 | 50.07 | 14 | -830 | NA | Style, Leaf |
| MD15G013510 | 57.01 | 15 | 67 | NA | Leaf |
| MD15G013780 | 608.44 | 15 | -307 | NADH dehydrogenase (ubiquinone) activity; oxidoreductase activity, acting on NAD(P)H, quinone or similar compound as acceptor | Leaf |
| MD15G029400 | 50.61 | 15 | -634 | NA | Leaf |
| MD15G035710 | 50.39 | 15 | -366 | NA | Pollen, Leaf |
| MD16G001600 | 52.39 | 16 | -420 | NA | Leaf |
| MD16G014720 | 57.02 | 16 | -284 | NA | Leaf |
| MD16G016550 | 129.25 | 16 | -582 | NA | Pollen, Leaf |
| MD16G017280 | 69.24 | 16 | -801 | NA | Leaf |
| MD17G009080 | 56.87 | 17 | -294 | lipid metabolic process; oxidoreductase activity, acting on paired donors, with oxidation of a pair of donors resulting in the reduction of molecular oxygen to two molecules of water | Pollen, Leaf |
| MD01G000690 | 122.39 | 1 | 65 | NA | Leaf |
| MD01G004640 | 62.75 | 1 | -751 | NA | Leaf |
| MD01G018030 | 62.22 | 1 | -690 | protein binding; signal transduction; cellular response to stimulus | Leaf |
| MD02G009650 | 69.61 | 2 | -421 | ubiquitin ligase complex；MdSFBB9-alpha | Pollen, Leaf |
| MD02G021280 | 52.34 | 2 | -466 | NA | Leaf |
| MD04G000340 | 59.26 | 4 | -649 | NA | Leaf |
| MD04G001150 | 80.38 | 4 | -664 | NA | Leaf |
| MD04G015280 | 60.06 | 4 | -229 | NA | Pollen, Leaf |
| **Plaza number** | **Peak score** | **Chr.** | **Distance.to.TSS** | **Predict function** | **Expressed tissues** |
| MD05G021280 | 55.7 | 5 | -125 | NA | Leaf |
| MD05G022730 | 52.05 | 5 | -573 | NA | Leaf |
| MD06G000750 | 66.33 | 6 | -883 | NA | Pollen, Leaf |
| MD06G005920 | 53.4 | 6 | -562 | NA | Leaf |
| MD06G011830 | 59.11 | 6 | -697 | integral to membrane; integral to membrane | Style, Leaf |
| MD07G007400 | 50.45 | 7 | -640 | NA | Leaf |
| MD07G016090 | 67.72 | 7 | -391 | heat shock protein binding; | Leaf |
| MD08G009340 | 84.38 | 8 | -626 | NA | Pollen, Leaf |
| MD09G007680 | 61.75 | 9 | -421 | NA | Leaf |
| MD09G014020 | 75.58 | 9 | 99 | NA | Leaf |
| MD09G027620 | 50.7 | 9 | -653 | NA | Pollen, Leaf |
| MD09G028020 | 68.61 | 9 | 31 | NA | Style, Leaf |
